# Exposure to Frontline Antiretroviral Dolutegravir Disrupts Oligodendrocyte Development Across Differentiation Stages

**DOI:** 10.1080/17590914.2026.2647877

**Published:** 2026-03-29

**Authors:** Marisa A. Jeffries, Raj Putatunda, Melanie Cruz-Berríos, Micah A. Romer, Anushka Singhal, Kelly L. Jordan-Sciutto, Judith B. Grinspan

**Affiliations:** a Division of Neurology, Children’s Hospital of Philadelphia, Philadelphia, PA, USA; b Perelman School of Medicine, University of Pennsylvania, Philadelphia, PA, USA

**Keywords:** Atf4, dolutegravir, integrated stress response, oligodendrocyte, Trib3

## Abstract

The use of antiretroviral (ART) treatment during pregnancy has dramatically reduced rates of perinatally-acquired human immunodeficiency virus 1 (HIV-1) infection to <1% in the United States. Despite this success, we have limited knowledge of how ART drugs that cross the placental barrier affect fetal development, particularly in the central nervous system (CNS). During gestation, large populations of oligodendroglia are produced that are responsible for critical postnatal CNS myelination enabling appropriate neurological function. Previous studies have shown that antiretrovirals impair oligodendrocyte (OL) differentiation leading us to hypothesize that OL maturation might be inhibited by exposure to a frontline ART drug cocktail (Triumeq®) prescribed during pregnancy containing dolutegravir (DTG), abacavir (ABC), and lamivudine (3TC). In this study, we demonstrated that exposing primary rat oligodendrocyte precursor cells (OPCs) and OLs to the Triumeq drug combination decreased OL maturation and myelin protein production in a concentration-dependent manner, and that DTG was solely responsible. Regardless of the timing of exposure during OL development, a high concentration of DTG inhibited OL maturation. Bulk RNA sequencing revealed transcriptional changes after DTG exposure related to a variety of cellular mechanisms, including cellular responses to stress pathways, amino acid starvation, and mitochondrial dysfunction. Although we found that DTG robustly activated the integrated stress response (ISR), attempted rescue experiments showed that DTG primarily inhibits OL maturation independently of the ISR. Collectively, our novel data on DTG underscore the necessity of investigating how ART drugs that are administered during pregnancy and cross the placental barrier can affect fetal CNS development.

## Introduction

Despite significant advances in medical treatment, human immunodeficiency virus 1 (HIV-1) infection continues to be a global healthcare burden. Approximately 40 million people worldwide are living with HIV-1 infection, with nearly 15% of infected individuals in the United States unaware of their medical condition. Of these 40 million people, the World Health Organization estimates 1.3 million women living with HIV become pregnant each year. Without intervention, 15-45% of these pregnancies result in vertical transmission of HIV-1 to the baby (Barral et al., 2014). However, the use of antiretroviral therapy (ART) during pregnancy has drastically reduced the rate of perinatal transmission to <1% in the United States (Lampe et al., 2023). Given the importance of ART in preventing vertical transmission and their ability to cross the placental barrier, data on the safety and efficacy of ART drugs on the developing CNS are needed. Current guidelines recommend the use of dolutegravir (DTG)-based regimens as preferred ART treatments during pregnancy, with few available alternatives having been rigorously tested for safety and efficacy. Previously, DTG came under intense international scrutiny after an observational study in Botswana demonstrated an eightfold increased risk for neural tube defects (NTDs) (Zash et al., 2018) in live births after perinatal exposure to DTG-based ART regimens. In contrast, other observational studies in Brazil (Pereira et al., 2021) and the United States (Reefhuis et al., 2020) did not demonstrate a correlation between DTG use at the time of pregnancy and NTDs. The unresolved concerns surrounding the safety of DTG exposure during central nervous system (CNS) development highlight the urgent need for additional research into the safety and efficacy of antiretroviral drugs taken during pregnancy.

Outside of pregnancy, what do we know about the impact of ART drugs on CNS function in people with HIV-1 (PWH)? ART treatment has reduced viral loads and increased life expectancies in PWH; however a subset of individuals present with a spectrum of cognitive and psychiatric abnormalities that affect 30-50% of PWH, termed HIV-1 associated neurocognitive disorders (HAND) (Heaton et al., 2010). HAND includes clinical classifications ranging from asymptomatic neurocognitive impairment (ANI), mild neurocognitive disorders (MND), to HIV-1 associated dementia (HAD). While ART has significantly decreased the prevalence of HAD, the incidence of ANI and MND has increased and overall HAND incidence in the pre- and post-ART eras are comparable (Heaton et al., 2010). Why does HAND persist despite effective viral suppression by ART? Unfortunately, growing evidence suggests that ART drugs themselves may contribute to CNS dysfunction (Lanman et al., 2021). Several *in vitro* studies have demonstrated that neurons are targets of antiretroviral neurotoxicity through mechanisms such as integrated stress response (ISR) activation and oxidative/organellar stress (Akay et al., 2014; Stern et al., 2018). Additionally, our research group has implicated ART drugs from the integrase inhibitor (INSTI) and protease inhibitor (PI) classes in disrupting oligodendrocyte (OL) maturation *in vitro* and *in vivo* through ISR activation (Roth et al., 2021), lysosomal de-acidification (Festa et al., 2021; 2023), and oxidative stress (Jensen et al., 2015). These specialized glial cells called OLs are generated by oligodendrocyte precursor cells (OPCs) and are responsible for producing and maintaining myelin in white matter throughout the CNS. Myelin, once thought to solely enable saltatory conduction of action potentials, is now understood to critically fine-tune CNS function, promote neural plasticity, and provide essential trophic and metabolic support to axons (Fünfschilling et al., 2012; Hirrlinger & Nave, 2014; Xin & Chan, 2020). Therefore, these data are particularly relevant as studies suggest white matter abnormalities are sustained in the CNS of PWH even with viral suppression by ART (Ackermann et al., 2014; Jankiewicz et al., 2017; Kelly et al., 2014; Tate et al., 2011).

Before the introduction of ART, white matter damage in PWH was quite robust, consisting of myelin pallor, extensive gliosis, and leukoencephalopathies (Everall et al., 2005; Gray et al., 1996). Even with ART adherence, studies have demonstrated that myelin and white matter disruptions persist in both adult (Gongvatana et al., 2011; Hoare et al., 2010) and pediatric (Ackermann et al., 2020; Graham et al., 2020; van Genderen et al., 2020) populations of PWH, with pathology severity directly correlating with both duration of ART treatment and neurocognitive status (Alakkas et al., 2019; Jernigan et al., 2011). Additionally, transcriptomic studies examining the frontal cortices of virally suppressed PWH with HAND showed dramatic decreases in specific RNA transcripts that encode proteins necessary for OL maturation and myelin maintenance and form a transcriptomic signature for HAND, such as myelin basic protein (*Mbp*), myelin-associated oligodendrocyte basic protein (*Mobp*), myelin transcription factor 1 (*Myt1*), myelin oligodendrocyte glycoprotein (*Mog*), and myelin associated glycoprotein (*Mag*) (Borjabad et al., 2010; Solomon et al., 2020), emphasizing a need to understand the impact of ART drugs on myelin development and homeostasis.

In this study, we focused on an ART drug cocktail, Triumeq, that is a frontline regimen for pregnant women with HIV-1 in the United States, Canada, and the European Union: an INSTI, DTG, and two nucleoside reverse transcriptase inhibitors (NRTIs), abacavir (ABC) and lamivudine (3TC). While the clinical evidence linking prenatal DTG exposure to NTDs is inconclusive, it is essential to elucidate any deleterious effects DTG-based regimens may have on the developing CNS, especially since current medical recommendations promote the use of DTG regimens during all stages of gestation. Given that ART-adherent pediatric PWH exhibit white matter alterations (Ackermann et al., 2020; Graham et al., 2020; van Genderen et al., 2020), we hypothesized that exposure to this ART cocktail affects the ability of OPCs to effectively differentiate into mature OLs. To address our hypothesis, we utilized primary OPC cultures from postnatal day 0–4 (P0–4) rat pups, a developmental stage equivalent to the third trimester in human gestation when OPCs are rapidly beginning to differentiate into mature OLs (Clancy et al., 2007; van Tilborg et al., 2018; Zeiss, 2021). Our data using primary OPC cultures reveal that exposure of oligodendroglia to DTG, but not ABC or 3TC, strongly inhibited OL differentiation *in vitro* in a dose-dependent manner. Regardless of whether exposure occurred during OPC proliferation or OL differentiation, a high concentration of DTG impaired OL maturation *in vitro*. We also determined that DTG exposure results in transcriptional changes in OLs related to cellular mechanisms such as stress responses and mitochondrial dysfunction. Our study is highly pertinent as all three ART drugs in this cocktail readily diffuse across the placental barrier from the umbilical cord at levels similar to plasma concentrations (Benaboud et al., 2012; Chappuy et al., 2004; Mulligan et al., 2018; Waitt et al., 2019), and could therefore impact the generation of OPC and OL populations in the developing fetal brain and their ability to form functional white matter.

## Methods

### Primary Rat OPCs

All experimental procedures involving live rats were approved by and performed in accordance with guidelines laid out by the Children’s Hospital of Philadelphia Institutional Animal Care and Use Committee. The isolation and culturing of primary rat OPCs was performed according to previously established protocols (Festa et al., 2021; Roth et al., 2021; Roth et al., 2021). Cerebral cortices were dissected out of P0-P4 Sprague Dawley rat pups, then mechanically dissociated before incubation in 0.25% trypsin (ThermoFisher Scientific, 15090046). Following filtration through a 100 μm mesh strainer, the cell pellet was resuspended in neurobasal medium (ThermoFisher Scientific, 21103049) containing B-27 supplement (ThermoFisher Scientific, 17504044), Pen/Strep (Corning, MT30-002-CI), and L-Glutamine (Corning, MT25-005-CI), and plated onto poly-D-lysine (Sigma, P6407) coated T-75 flasks. The neurobasal/B-27 medium was changed after 24 hours to OPC growth medium (Neurobasal/B-27 medium supplemented with bovine fibroblast growth factor (bFGF; 10 ng/mL; R&D Systems, 133-FB-025), recombinant human platelet-derived growth factor (PDGF-AA; 2 ng/mL; R&D Systems, 221AA025), and neurotrophin 3 (NT3; 1 ng/mL; ThermoFisher Scientific, 450-03-10UG) (Festa et al., 2021; Roth et al., 2021; Roth et al., 2021)). Once the mixed glial cell culture reached 90% confluency, a shake-off protocol was performed to increase the purity of the OPC cultures by up to 95% (McCarthy & de Vellis, 1980). T-75 flasks were incubated at 37 °C for 1 hour on an orbital shaker set to 250 RPM to detach microglia followed by an overnight incubation at the same settings. Medium containing the detached OPCs was filtered through a 20 μm mesh strainer, and then centrifuged for 5 minutes at 1500 RPM at 4 °C. The cell pellet was resuspended in Neurobasal/B-27 medium and plated onto a bacteriological petri dish then incubated at 37 °C/5% CO_2_ for 15 minutes. Medium was collected and centrifuged for 5 minutes at 1500 RPM at 4 °C, the cell pellet was resuspended in OPC growth medium and plated onto poly-D-lysine-coated culture plasticware.

### *In Vitro* Differentiation of OPCs and Antiretroviral Drug Treatments

Purified rat OPCs were cultured to 70-90% confluency on 12 mm coverslips in 24-well plates for immunocytochemistry (ICC) or 10 cm tissue culture dishes for Western blotting protein collection. Once adequate OPC cell confluency was reached, OPCs were differentiated into mature OLs for 72 hours by switching from OPC proliferation medium to OPC differentiation medium consisting of DMEM/F12, Penicillin/Streptomycin, 2 mM L-glutamine, 50 μg/mL transferrin, 5 μg/mL putrescine, 3 ng/mL progesterone, 2.6 ng/mL selenium, 12.5 μg/mL insulin, 0.5 μg/mL thyroxine, 0.3% glucose, and 10 ng/mL biotin (Festa et al., 2021; Jensen et al., 2015; Roth et al., 2021; Roth et al., 2021). In this study, OPCs were treated with the antiretroviral drugs DTG (Toronto Research Chemicals, D528800 and MedChemExpress, HY-13238), ABC (MedChemExpress, HY-17423), 3TC (MedChemExpress, HY-B0250), or a combination of all 3 drugs (identified as cART). The specific doses tested in this study are delineated in Table 1 and were chosen to cover a range surrounding human plasma C_max_ of each drug (Cottrell et al., 2013; Else et al., 2012; Wang et al., 1999). Human plasma C_max_ represents the peak concentration of the drug in the periphery, and may not accurately reflect concentrations within the CNS. However, while cerebrospinal fluid (CSF) levels of ART drugs are reportedly lower than those observed in serum, ART drug levels in brain tissues have been shown to be higher than in CSF (Ferrara et al., 2020; Srinivas et al., 2019), and some ART drugs accumulate in white matter regions where OPCs and OLs are present at high densities (Ferrara et al., 2020). Additionally, all three drugs (DTG, ABC, 3TC) cross the placenta at levels nearly equal to C_max_ (Best et al., 2006; Chappuy et al., 2004; Moodley et al., 1998; Mulligan et al., 2018; Waitt et al., 2019), although the efficiency of drug transfer across the developing blood brain barrier (BBB) in the fetus is not known. With this in mind, we chose to investigate doses at 10% of C_max_ at the lower end as well as 300% of C_max_ at the higher end. Multistage ART drug treatment during both OPC proliferation and OL maturation was performed to closely mimic gestational expansion of OPCs and early OL differentiation. We also treated cultures only during either proliferation or differentiation to elucidate stage-specific effects of DTG exposure. For all cell culture experiments, 10 µM elvitegravir was used as a validation control for impaired OL differentiation and all replicates showed appropriate response to this drug as observed in Roth et al. (2021). Thapsigargin treatment (500 nM for 2 hours) was used as a positive control for integrated stress response activation experiments and worked to robustly increase phosphorylated eIF2α protein levels (data not shown).

**Table 1.** Antiretroviral drug doses tested in this study, corresponding to the plasma C_max_ levels that were reported in the literature.

Antiretroviral compound	Low dose (10% of C_max_)	Middle dose (C_max_)	High dose (300% C_max_)	C_max_ reference
Dolutegravir (DTG) – INSTI	800nM	8μM	24μM	(Cottrell et al., 2013)
Abacavir (ABC) – NRTI	150nM	1.5 μM	4.5 μM	(Wang et al., 1999)
Lamivudine (3TC) – NRTI	600nM	6μM	18μM	(Else et al., 2012)

### Immunocytochemistry

Primary antibodies mouse anti-A2B5 (1:2 in DMEM/F12, IgM hybridoma supernatant (Eisenbarth et al., 1979)), mouse anti-O4 (1:2 in DMEM/F12, IgM hybridoma supernatant (Sommer & Schachner, 1981)), mouse anti-GalC (1:5 in DMEM/F12, IgG_3_ hybridoma supernatant (Raff et al., 1978)) and rat anti-PLP (1:2 in 1x PBS, IgG hybridoma supernatant AA3, a gift from Alex Gow at Wayne State University (Roth et al., 2021)) were prepared in-house and diluted as indicated. Their corresponding host-specific fluorescently-tagged secondary antibodies Alexa-Fluor 647 conjugated goat anti-mouse IgM (1:200, Jackson ImmunoResearch, 115-605-075, RRID: AB_2338911), Rhodamine (TRITC) conjugated goat anti-mouse IgM (1:200, Jackson ImmunoResearch, 115-025-075, RRID: AB_2338487), Alexa-Fluor 488 conjugated goat anti-mouse IgG_3_ (1:1000, Southern Biotech, 1100-30, RRID: AB_2794582), Rhodamine-conjugated goat anti-rat IgG (1:200, Jackson ImmunoResearch, 112-025-062, RRID: AB_2338116), and FITC conjugated goat anti-mouse IgG (1:200, Jackson ImmunoResearch, 115-095-003, RRID: AB_2338589) were diluted similarly. Cell culture coverslips were incubated at room temperature for 30 minutes with cell surface primary antibodies for mouse anti-A2B5 for OPCs, mouse anti-O4 for early OLs, or mouse anti-GalC for mature OLs followed by a 30 minute incubation with secondaries: Alexa-Fluor 647 conjugated goat anti-mouse IgM, Alexa-Fluor Cy3 conjugated goat anti-mouse IgM, FITC conjugated goat anti-mouse IgG, or Alexa-Fluor 488 conjugated goat anti-mouse IgG_3_. After fixation in ice-cold 100% methanol for 7–10 minutes some coverslips were incubated at room temperature for 30 minutes with rat anti-PLP followed by a 30 minute incubation in Rhodamine-conjugated goat anti-rat IgG. Cells were stained with DAPI (1:10000 in 1X PBS, Fisher Scientific, D1306) for 5 minutes then coverslips were mounted onto glass slides with Prolong Gold Antifade Mounting Reagent (ThermoFisher Scientific, P36930, RRID:SCR_015961). For ATF4 immunostaining, our previously published protocol was used (Roth et al., 2021). Briefly, cells were fixed in ice cold 4% paraformaldehyde for 10 minutes then permeabilized for 30 minutes in 0.1% Triton X-100 in 1x PBS, then blocked at room temperature for 30 minutes in 10% normal goat serum in 1x PBS. Coverslips were incubated at 4 °C overnight with rabbit anti-ATF4 antibody (1:100 in 1x PBS with 5% normal goat serum, Proteintech, 10835-1-AP, RRID: AB_2058600) overnight at 4 °C. The next day, the cells were washed and incubated with Rhodamine Red-conjugated goat anti-rabbit secondary antibody (1:200 in 5% normal goat serum/1x PBS, Jackson ImmunoResearch, 111-295-003, RRID: AB_2338022) for 30 minutes at room temperature. Coverslips were incubated in DAPI, and mounted onto glass microscope slides as described above. All coverslips were imaged at 40x or 100x magnifications using Keyence BZ-X-700 or BZ-X-800 digital fluorescent microscopes (Keyence, RRID: SCR_016979, RRID: SCR_023617) or DMi8 Leica inverted confocal microscope (Leica Microsystems).

### TUNEL Assay

To assess for late-stage apoptotic cell death, we used a modified TUNEL staining protocol. First, differentiated OLs were fixed with 4% paraformaldehyde for 15 minutes, then permeabilized with 0.1% Triton X-100 and 0.5% BSA in 1X PBS for 30 minutes. Positive control coverslips were generated by incubating in DN buffer (30 mM Trizma base pH 7.2, 140 mM sodium cacodylate, 4 mM magnesium chloride, and 0.1 mM dithiothreitol) for 2 minutes, followed by a 10-minute incubation with DNAse (1:200 in DN buffer). Coverslips were incubated with TdT buffer (30 mM Trizma base pH 7.2, 140 mM sodium cacodylate, and 1 mM cobalt chloride) for 2 minutes, followed by a 1 hour incubation at 37 °C with TdT (Roche, 3333566001) and biotin-UTP (Roche, 11093070910) (6 μL of each dissolved in 1 mL of TdT buffer). After washing, cells were incubated at room temperature for 15 minutes in TB buffer (300 mM sodium chloride and 30 mM sodium citrate) and 30 minutes in 2% bovine serum albumin (BSA) solution, then 30 minutes with Rhodamine-conjugated streptavidin secondary antibody (1:200 in 1x PBS, Jackson ImmunoResearch, 016-290-084, RRID: AB_2337247). After incubation with DAPI (1:10000 in 1x PBS) for 5 minutes, coverslips were mounted onto glass microscope slides and imaged as described above. For experiments examining late-stage apoptosis in OL cultures with extended exposure to DTG, a CoraLite 594 TUNEL Assay Apoptosis Detection Kit (Proteintech, PF00009) was utilized. Briefly, coverslips were fixed in 4% paraformaldehyde for 30 minutes at 4 °C, then permeabilized in 0.2% Triton X-100 for 20 minutes. Positive control coverslips were generated by incubating coverslips in kit-provided 1x DNase buffer for 5 minutes followed by kit-provided 1:100 DNase I (in DNase buffer) for 10 minutes. Coverslips were then incubated with kit-provided TdT enzyme in CoraLite 594 TdT reaction buffer for 60 minutes at 37 °C. After washing, coverslips were incubated 3x for 5 minutes in 0.1% Triton X-100 + 5 mg/mL BSA in 1x PBS, counterstained with DAPI (1:10000 in 1x PBS) for 10 minutes, and mounted onto glass microscope slides.

### Protein Collection and Western Blotting

Whole-cell protein extracts from differentiated OLs were prepared in protein lysis buffer (25 mM Tris-HCl pH 7.4, 10 mM EDTA, 10% SDS, 1% Triton X-100, and 150 mM NaCl) containing cOmplete mini, EDTA-free protease inhibitor cocktail (Roche, 11836170001) and PhosSTOP phosphatase inhibitor (Roche, 4906845001) with sonication then centrifuged at 14000 RPM for 20 minutes at 4 °C. Protein concentrations in the supernatant lysates were determined using the Pierce BCA Protein Assay Kit (ThermoFisher Scientific, 23227). Equal amounts of protein (10 μg for myelin protein blots, 20 μg for phosphorylated eukaryotic initiation factor 2-alpha [p-eIF2α]/total eukaryotic initiation factor 2-alpha [t-eIF2α] protein blots) were electrophoretically resolved on 4-12% Bis-Tris gradient gels for 55 minutes at 180 V, then transferred onto Immobilion-FL PVDF membranes for 1 hour at 30 V. After the transfer, membranes were blocked at room temperature for 30 minutes in either 5% milk in TBST or 5% BSA in TBST. Membranes were then probed with specific primary antibodies overnight at 4 °C. Primary antibodies to the following proteins were utilized: mouse anti-MBP (1:1000, Clone SMI-99, Biolegend, 808401, RRID: AB_2564741), rat anti-PLP (1:1000, rat hybridoma), rabbit anti-phosphorylated-eIF2α (Ser 51) (1:1000, Cell Signaling, 9721, RRID: AB_330951), mouse anti-total-eIF2α (1:1000, Cell Signaling, 2103, RRID: AB_213690), and mouse anti-α-Tubulin (1:20000, Sigma, T5168, RRID: AB_477579). Membranes were incubated at room temperature for 1 hour with corresponding host-specific fluorescently-tagged secondary antibodies (all diluted 1:10000 in TBST with 5% BSA). The following secondary antibodies were utilized: goat anti-mouse IRdye 680RD (LI-COR Biosciences, 925-68,070, RRID: AB_2651128), goat anti-rat IRdye 680RD (LI-COR Biosciences, 926-32,229, RRID: AB_1850020), goat anti-mouse IRdye 800CW (LI-COR Biosciences, 926-32,212, RRID: AB_621847), and goat anti-rabbit IRdye 680RD (LI-COR Biosciences, 926-68,071, RRID: AB_10956166). After secondary antibody incubation, membranes were imaged on an Odyssey 9120 infrared imaging system (LI-COR Biosciences). Densitometric analyses were performed using FIJI (NIH, RRID: SCR_002285).

### RNA Sequencing: Library Preparation with PolyA Selection and Illumina Sequencing

RNA was isolated from cultured OPCs or OLs using the Qiagen RNeasy Mini Kit (Qiagen, 74104) then concentration measured by Nanodrop One Spectrophotometer (Thermo Scientific) before shipment to Azenta Life Sciences for sequencing. At Azenta, total RNA samples were quantified using Qubit 2.0 Fluorometer (Life Technologies) and RNA integrity was checked using Agilent TapeStation 4200 (Agilent Technologies). ERCC RNA Spike-In Mix (ThermoFisher Scientific, 4456740) was added to normalized total RNA prior to library preparation following manufacturer’s protocol. RNA sequencing libraries were prepared using the NEBNext Ultra II RNA Library Prep Kit for Illumina using manufacturer’s instructions (NEB). Briefly, mRNAs were initially enriched with Oligod(T) beads. Enriched mRNAs were fragmented for 15 minutes at 94 °C. First strand and second strand cDNA were subsequently synthesized. cDNA fragments were end repaired and adenylated at 3′ ends, and universal adapters were ligated to cDNA fragments, followed by index addition and library enrichment by PCR with limited cycles. The sequencing library was validated on the Agilent TapeStation (Agilent Technologies), and quantified by using Qubit 2.0 Fluorometer (Invitrogen) as well as by quantitative PCR (KAPA Biosystems). The sequencing libraries were multiplexed and clustered onto a flowcell on the Illumina NovaSeq instrument according to manufacturer’s instructions. The samples were sequenced using a 2x150bp Paired End (PE) configuration. Image analysis and base calling were conducted by the NovaSeq Control Software (NCS). Raw sequence data (.bcl files) generated from Illumina NovaSeq was converted into fastq files and de-multiplexed using Illumina bcl2fastq 2.20 software. One mis-match was allowed for index sequence identification.

### RNA Sequencing: Data Analysis

After investigating the quality of the raw data, sequence reads were trimmed to remove possible adapter sequences and nucleotides with poor quality. The trimmed reads were mapped to the reference genome available on ENSEMBL using the STAR aligner v.2.5.2b. The STAR aligner is a splice aligner that detects splice junctions and incorporates them to help align the entire read sequences. BAM files were generated as a result of this step. Unique gene hit counts were calculated by using feature Counts from the Subread package v.1.5.2. Only unique reads that fell within exon regions were counted. After extraction of gene hit counts, the gene hit counts table was used for downstream differential expression analysis. Using DESeq2, a comparison of gene expression between the groups of samples was performed. The Wald test was used to generate p-values and Log2 fold changes. Genes with adjusted *p*-values < 0.05 and absolute log2 fold changes > 1 were called as differentially expressed genes for each comparison. A gene ontology analysis was performed on the statistically significant set of genes by implementing the software GeneSCF. The human GO list was used to cluster the set of genes based on their biological process and determine their statistical significance. A PCA analysis was performed using the “plotPCA” function within the DESeq2 R package. The plot shows the samples in a 2D plane spanned by their first two principal components. The top 500 genes, selected by highest row variance, were used to generate the plot.

### qRT-PCR

Purified RNA was isolated from cultured OPCs or OLs using the Qiagen RNeasy Mini Kit (Qiagen, 74104). cDNA was synthesized using the Invitrogen SuperScript III First-Strand Synthesis System for RT-PCR (Invitrogen, 18080-051) and the Eppendorf Mastercycler Pro Model 6321. For each qRT-PCR reaction, *Pgk1* was used as a housekeeping gene for quantification. For *Trib3,* the Rn06210329_s1 Taqman gene expression assay (ThermoFisher Scientific, 4448892) and the *Pgk1* Rn01474008_gH Taqman gene expression assay (ThermoFisher Scientific, 4448892) were used along with Taqman Fast Advanced Master Mix (ThermoFisher Scientific, 4444556). For *Mbp, Atf4,* and *Cebpb***,** PowerUp SYBR Green Master Mix for qPCR (ThermoFisher Scientific, A25742) was used with the following primers: *Mbp* forward, 5′-TGAAAACCCAGTAGTCCAC-3′; *Mbp* reverse, 5′-GGATTAAGAGAGGGTCTGC-3′; *Atf4* forward, 5′-GTTGGTCAGTGCCTCAGACA-3′; *Atf4* reverse, 5′-CATTCGAAACAGAGCATCGA-3′; *Cebpb* forward, 5′-ACGACTTCCTTTCCGACCTC-3′; *Cebpb* reverse, 5′-ACGTAACCGTAGTCGGACG-3′; *Pgk1* forward, 5′-ATGCAAAGACTGGCCAAGCTAC-3′; *Pgk1* reverse, 5′-AGCCACAGCCTCAGCATATTTC-3′. Samples were loaded in triplicate and all qRT-PCR experiments were run on a QuantStudio 7 Flex Real-Time PCR System (ThermoFisher Scientific, RRID: SCR_020245).

### Data Quantification and Statistical Analysis

For all experiments, primary OPCs prepared from independent litters were designated as separate biological replicates. All cell counts and Western blotting densitometry data are represented as the Mean ± SEM and normalized to an Untreated (UT) group that has not been treated with a vehicle or drug, as has been done previously (Festa et al., 2021; Jensen et al., 2015; Roth et al., 2021; Roth et al., 2021). All differentiation cell counts were performed from at least 3 independent biological replicates, consisting of 2–4 coverslips per treatment group, with cell counts performed on 10–25 random 40x fields per coverslip. For ATF4 immunocytochemistry in proliferation and differentiation experiments, confocal images were acquired using a 40x oil objective, and ATF4 nuclear staining intensity was quantified using integrated density in FIJI. For qRT-PCR data, all samples were run in triplicate; for any sample showing >0.5 standard deviation for Ct values, the Ct value with the greatest difference was excluded. This occurred in only one sample for *Pgk1* run with *Cebpb*. All statistical analyses were performed using GraphPad Prism (RRID: SCR_002798). Specifically, a one-way ANOVA was performed to determine statistically significant differences in experiments with more than two groups, with a *post-hoc* Dunnett multiple comparisons analysis to assess changes between all treatment conditions compared to vehicle controls. For the experiments using ISRIB, a one-way ANOVA with a *post-hoc* Sidak multiple comparisons analysis was used to assess changes between all treatment conditions compared to vehicle controls and between each dose of DTG and the corresponding dose of DTG + ISRIB. For experiments using only two groups, an unpaired t-test was used to determine statistical significance.

## Results

### The Triumeq Antiretroviral Drug Cocktail Inhibits Oligodendrocyte Differentiation and Myelin Protein Production in a Dose-Dependent Manner

**Figure 1.**
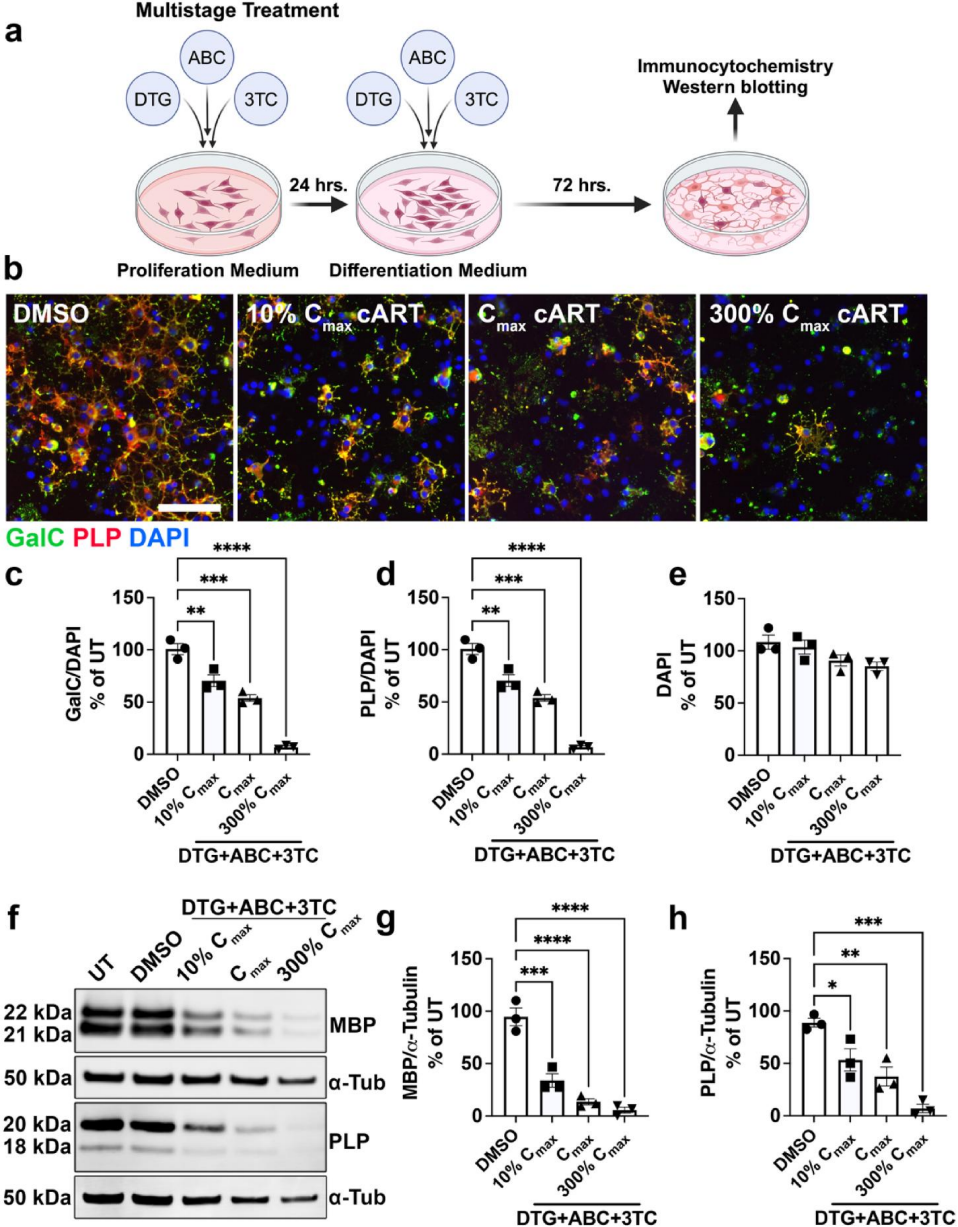
Multistage exposure to increasing doses of DTG+ABC + 3TC inhibits OL maturation. (a) Diagram of multistage treatment paradigm with DTG+ABC + 3TC during OPC proliferation and OL differentiation. (b) Representative images of differentiated OLs after multistage treatment with DTG+ABC + 3TC and immunostained for GalC (green), PLP (red), and DAPI (blue). Scale bar = 75 µm. Quantification of the number of GalC^+^ (c) and PLP^+^ (d) cells shows significantly fewer differentiated OLs after multistage exposure to DTG+ABC + 3TC without an effect on total number of DAPI^+^ cells (e), n = 3/group. **f)** Representative immunoblots of MBP and PLP after multistage exposure to DTG+ABC + 3TC. Densitometry analyses show significantly less MBP (g) and PLP (h) expression, *n* = 3/group. One-way ANOVA with Dunnett post-hoc test to compare all treatment groups to DMSO. *p < 0.05, **p < 0.01, ***p < 0.001, ****p < 0.0001.

Triumeq consists of the INSTI DTG combined with two NRTIs, ABC and 3TC, which are preferred antiretrovirals to treat HIV during pregnancy that cross the placental barrier (Benaboud et al., 2012; Chappuy et al., 2004; Mulligan et al., 2018; Waitt et al., 2019). Given that substantial oligodendroglial development occurs during gestation, we sought to determine whether this frontline drug combination affects differentiation of OPCs into OLs and myelin protein production by mature OLs. We treated primary rat OPCs *in vitro* with 3 doses of combined DTG+ABC + 3TC (cART) for 24 hours in the OPC stage, then changed to differentiation medium with the same doses of DTG+ABC + 3TC for 72 hours (Figure 1a). These 3 doses corresponded to 10% of human plasma C_max_ (800 nM DTG + 150 nM ABC + 600 nM 3TC), human plasma C_max_ (8 µM DTG + 1.5 µM ABC + 6 µM 3TC), and 300% human plasma C_max_ (24 µM DTG + 4.5 µM ABC + 18 µM 3TC) (Table 1, see methods). We selected doses surrounding C_max_ because all three drugs cross the human placental barrier at levels approximately equal to plasma C_max_ (DTG 1.25x, ABC 1x, and 3TC 0.86x) (Best et al., 2006; Chappuy et al., 2004; Moodley et al., 1998; Mulligan et al., 2018; Waitt et al., 2019), although it is unknown how efficiently they then cross the BBB in the developing fetus or whether they accumulate in developing white matter regions. This “multistage treatment” across both proliferation and differentiation models exposure to DTG during third trimester human fetal gestation when both robust OPC proliferation and OL differentiation occur (Clancy et al., 2007; van Tilborg et al., 2018; Zeiss, 2021). We observed that multistage treatment with 10% C_max_ cART, C_max_ cART, and 300% C_max_ cART resulted in significantly fewer OLs (Figure 1b) positive for either galactocerebrosidase (GalC) (Figure 1c) or proteolipid protein (PLP) (Figure 1d), markers of mature OLs, in a dose-dependent manner without affecting the number of DAPI+ cells (Figure 1e). We also assessed myelin protein levels in differentiated OL whole-cell lysates by Western blot (Figure 1f) and found that the DTG+ABC + 3TC drug combination resulted in significantly less MBP (Figure 1g) and PLP (Figure 1h) expression in differentiating OLs compared to DMSO, again in a dose-dependent manner. Taken together, these data demonstrate that exposure to increasing doses of combined DTG+ABC + 3TC significantly decreases OL differentiation and myelin protein production *in vitro*.

### Dolutegravir, but not Abacavir or Lamivudine, Inhibits Oligodendrocyte Differentiation and Myelin Protein Production

**Figure 2.**
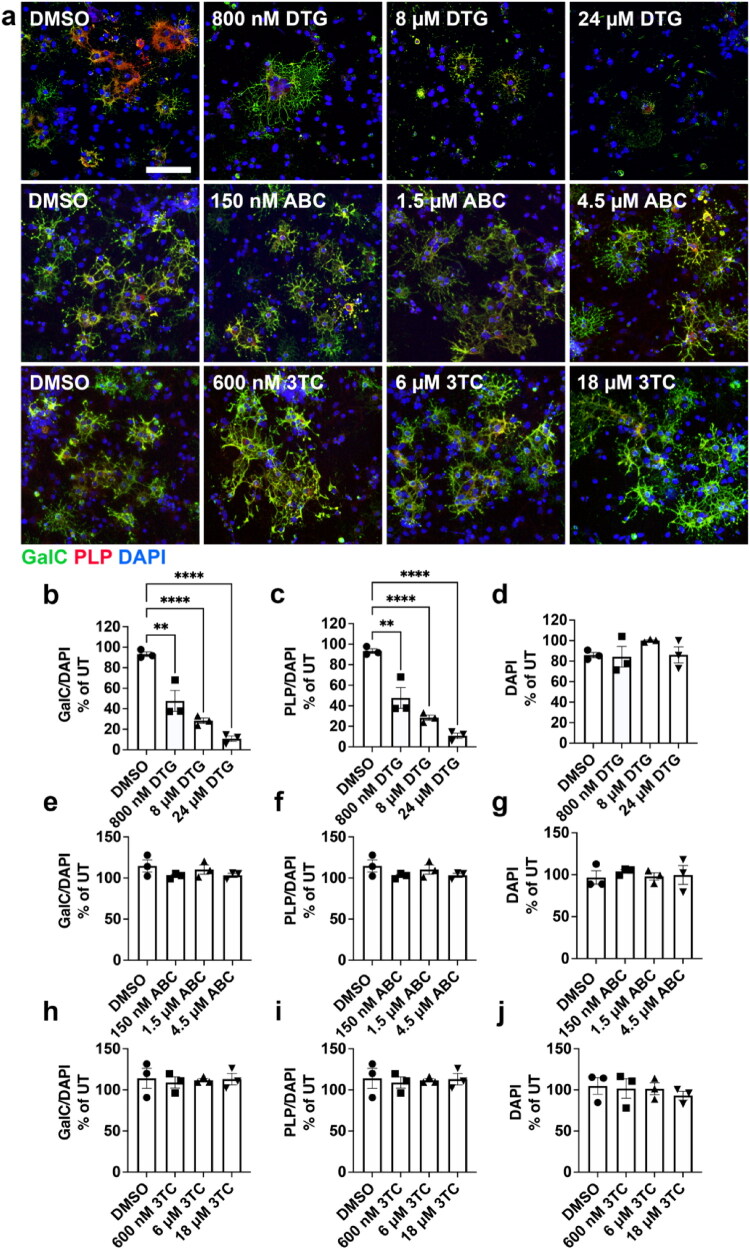
DTG, but not ABC or 3TC, significantly impairs OL maturation after multistage exposure. (a) Representative images of differentiated OLs after multistage treatment with DTG, ABC, or 3TC and immunostained for GalC (green), PLP (red), and DAPI (blue). Scale bar = 75 µm. Quantification of the number of GalC^+^ and PLP^+^ cells shows significantly fewer differentiated OLs without a change in DAPI after multistage exposure to DTG (b-d) but not ABC (e-g) or 3TC (h-j), *n* = 3/group. One-way ANOVA with Dunnett post-hoc test to compare all treatment groups to DMSO. **p < 0.01, ****p < 0.0001.

**Figure 3.**
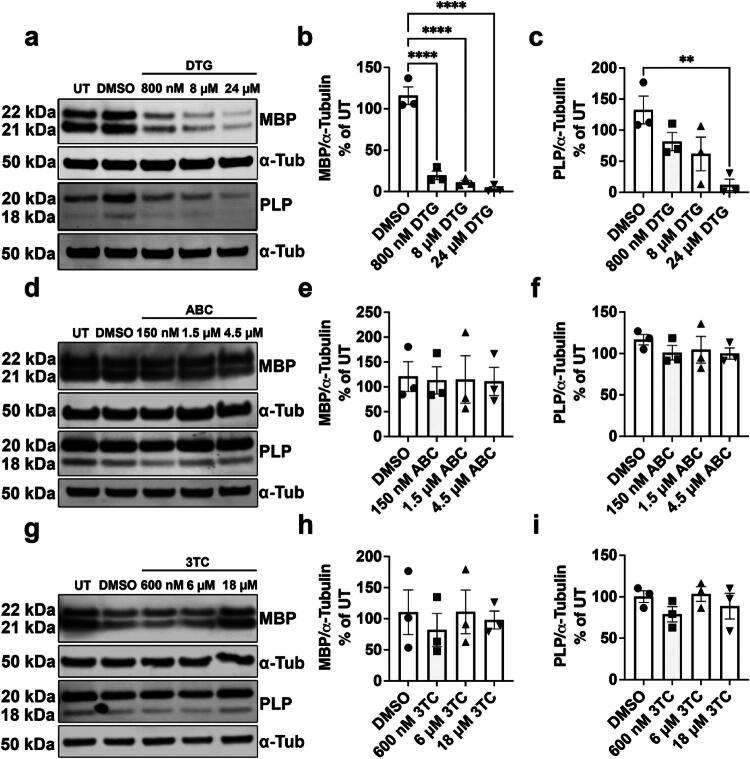
DTG, but not ABC or 3TC, significantly reduces myelin protein expression after multistage exposure. (a) Representative immunoblots of MBP and PLP after multistage exposure to DTG (800 nM, 8 µM, or 24 µM). Densitometry analyses show significantly less MBP after exposure to 800 nM, 8 µM, or 24 µM DTG (b) and PLP after exposure to 24 µM DTG (c), *n* = 3/group. (d) Representative immunoblots of MBP and PLP after multistage exposure to ABC (150 nM, 1.5 µM, or 4.5 µM). Densitometry analyses show no changes in MBP (e) or PLP (f) expression, *n* = 3/group. **g)** Representative immunoblots of MBP and PLP after multistage exposure to 3TC (600 nM, 6 µM, or 18 µM). Densitometry analyses show no changes in MBP (h) or PLP (i) expression, *n* = 3/group. One-way ANOVA with Dunnett post-hoc test to compare all treatment groups to DMSO. **p < 0.01, ****p < 0.0001.

Since the DTG+ABC + 3TC ART drug combination significantly decreased OPC differentiation and myelin protein levels, we next wanted to assess which specific drug(s) was responsible for this effect. Therefore, we treated primary OPCs individually with DTG (800 nM, 8 µM, or 24 µM), ABC (150 nM, 1.5 µM, or 4.5 µM), or 3TC (600 nM, 6 µM, or 18 µM) using the multistage treatment paradigm (Figure 2a). DTG treatment resulted in significantly lower numbers of GalC+ (Figure 2b) and PLP+ (Figure 2c) OLs compared to DMSO controls in a concentration-dependent manner without altering the number of DAPI+ cells in culture (Figure 2d). In contrast, neither ABC nor 3TC altered GalC+ (Figure 2e,h), PLP+ (Figure 2f,i), or DAPI+ (Figure 2g,j) cell counts. Our data reveal that DTG is the ART drug in the Triumeq combination primarily responsible for the observed deficit in OL differentiation. Western blots were subsequently performed to assess if DTG (Figure 3a), ABC (Figure 3d), and 3TC (Figure 3g) affected MBP and PLP protein levels and found that increasing doses of DTG resulted in significantly lower amounts of MBP (Figure 3b) and PLP (Figure 3c) protein levels compared to DMSO controls. Conversely, ABC did not alter MBP (Figure 3e) or PLP (Figure 3f) protein levels *in vitro*. Similarly, increasing concentrations of 3TC also did not alter MBP (Figure 3h) or PLP (Figure 3i) protein levels. These data demonstrate that in the Triumeq drug regimen, DTG is primarily responsible for the deficits in OL differentiation and myelin protein expression.

### Dolutegravir Does Not Induce Apoptosis During OL Differentiation

Since there was a robust decrease in OPC differentiation and myelin production in DTG-treated OPCs, we next assessed whether this effect was due to cellular apoptosis. To assess for apoptosis, 800 nM, 8 µM, or 24 µM DTG-treated primary rat OLs were labeled with terminal deoxynucleotidyl transferase dUTP nick end labeling (TUNEL), which is a histological marker for double-stranded DNA breaks (Supp. Fig. 1a). Statistical analysis revealed that increasing concentrations of DTG did not significantly alter numbers of TUNEL+ nuclei, despite a trend toward increased apoptosis in OLs exposed to 8 µM or 24 µM DTG (Supp. Fig. 1b). However, given the strong trend toward increased cell death with 8 µM and 24 µM DTG exposure, we also extended our treatment to 24 hours during proliferation plus 120 hours during differentiation to determine whether extended DTG exposure would be sufficient to significantly increase apoptosis. TUNEL labeling after 5 days of differentiation did not reveal any significant differences in the numbers of TUNEL+ nuclei (Supp. Fig. 1c-d), and we likewise did not observe any change in overall DAPI+ nuclei that would indicate cell loss (Supp. Fig. 1e). Taken together, these data demonstrate that cell death is not responsible for the significant DTG-mediated decreases in OL maturation and myelin protein production.

### Dolutegravir Specifically Impairs Late-Stage OL Maturation

Impaired OL differentiation in the presence of DTG could arise from reduced OPC numbers from which to differentiate. As OL differentiation occurs on a continuum and the expression of GalC and PLP occurs relatively late in maturation, we also sought to determine which stage of differentiation DTG was inhibiting. To assess the numbers of OPCs, cultures exposed to 800 nM, 8 µM, or 24 µM DTG were immunostained for the OPC marker A2B5 (Supp. Fig. 2a). After 72 hours of differentiation, the numbers of OPCs did not change following DTG treatment (Supp. Fig. 2b). Therefore, reduced OL differentiation in the presence of DTG was not due to a reduced pool of OPCs in culture. We next immunostained 800 nM, 8 µM, or 24 µM DTG-exposed cultures for O4, a marker of early OLs that continues to be expressed as cells mature, along with GalC (Supp Fig. 2c). Our quantification revealed no change in O4+ early OLs that were not yet GalC + in DTG-treated cultures compared to controls (Supp. Fig. 2d). Taken together, these data indicate that DTG specifically inhibits late-stage OL maturation, resulting in fewer mature GalC+/PLP+ OLs without affecting the number of A2B5+ OPCs or O4+/GalC- early OLs.

### Exposure to 24 µM DTG During Either OPC Proliferation or OL Differentiation is Sufficient to Impair Differentiation

**Figure 4.**
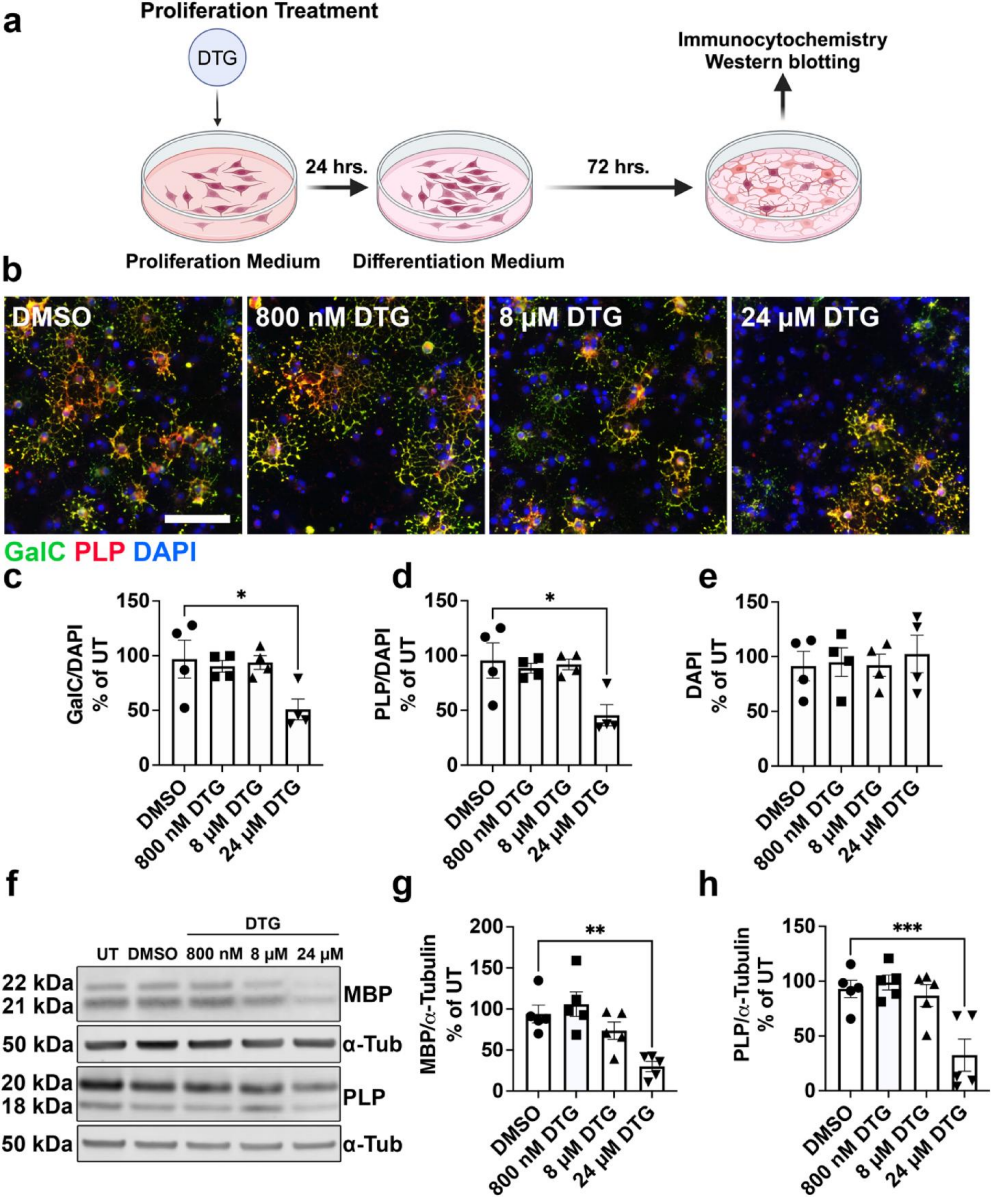
Treatment of proliferating OPCs with 24 µM DTG inhibits subsequent maturation. (a) Diagram of proliferation treatment paradigm with DTG during OPC proliferation only. (b) Representative images of differentiated OLs after proliferation treatment with DTG (800 nM, 8 µM, or 24 µM) and immunostained for GalC (green), PLP (red), and DAPI (blue). Scale bar = 75 µm. Quantification of the number of GalC^+^ (c) and PLP^+^ (d) cells shows significantly fewer differentiated OLs after proliferation exposure to 24 µM DTG without an effect on total number of DAPI^+^ cells (e), *n* = 4/group. (f) Representative immunoblots of MBP and PLP after proliferation exposure to DTG. Densitometry analyses show significantly less MBP (g) and PLP (h) expression after 24 µM DTG exposure, *n* = 5/group. One-way ANOVA with Dunnett post-hoc test to compare all treatment groups to DMSO. *p < 0.05, **p < 0.01, ***p < 0.001.

**Figure 5.**
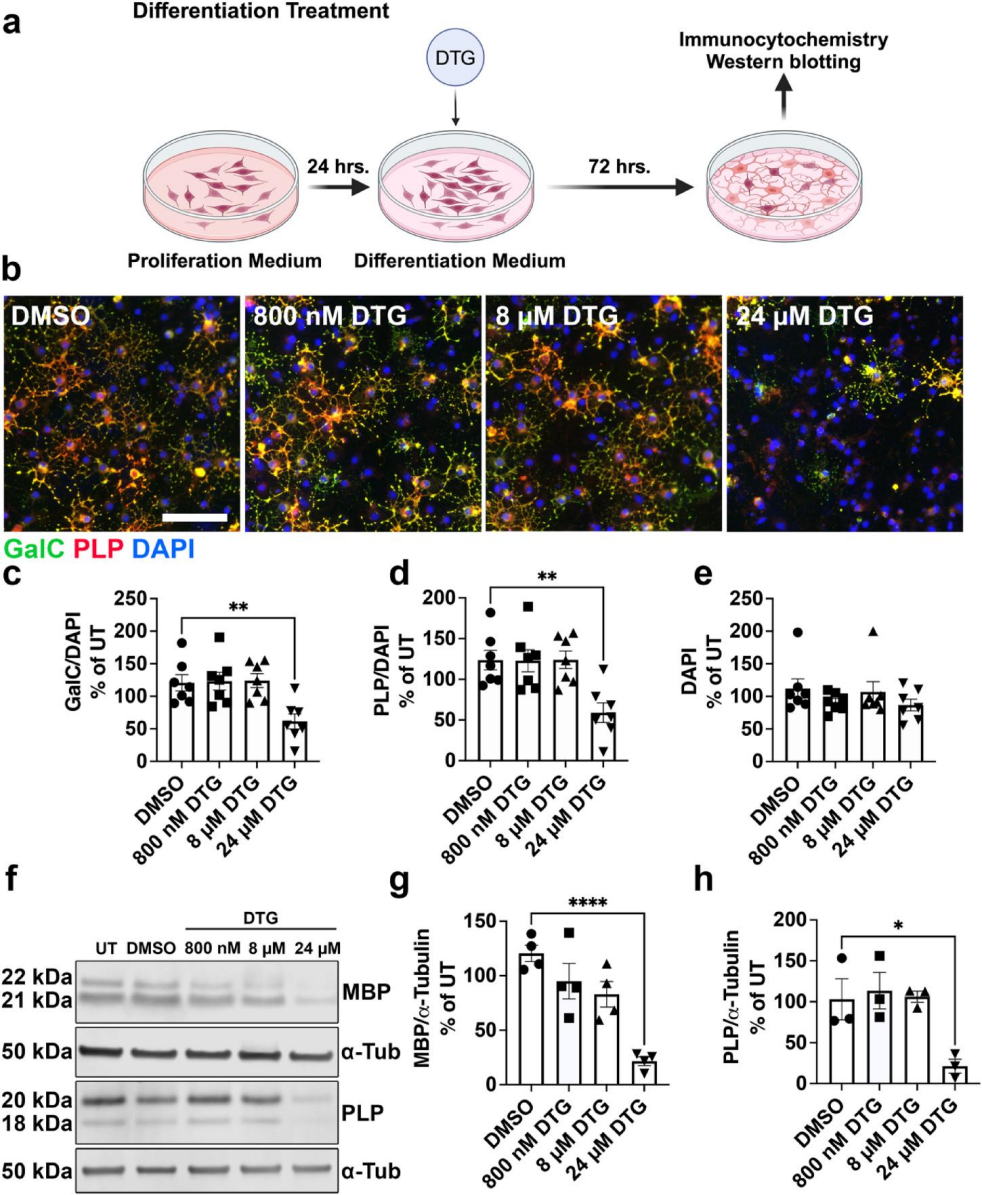
Treatment of OLs during differentiation with 24 µM DTG reduces OL maturation. (a) Diagram of differentiation treatment paradigm with DTG during OL differentiation only. (b) Representative images of differentiated OLs after differentiation treatment with DTG (800 nM, 8 µM, or 24 µM) and immunostained for GalC (green), PLP (red), and DAPI (blue). Scale bar = 75 µm. Quantification of the number of GalC^+^ (c) and PLP^+^ (d) cells shows significantly fewer differentiated OLs after differentiation exposure to 24 µM DTG without an effect on total number of DAPI^+^ cells (e), *n* = 7/group. (f) Representative immunoblots of MBP and PLP after differentiation exposure to DTG. Densitometry analyses show significantly less MBP (g) and PLP (h) expression after 24 µM DTG exposure, *n* = 3-4/group. One-way ANOVA with Dunnett post-hoc test to compare all treatment groups to DMSO. *p < 0.05, **p < 0.01, ****p < 0.0001.

Given the remarkable effect of DTG on OL differentiation when applied across proliferation and differentiation, we next wanted to determine whether the reduction in OL maturation was due to exposure during proliferation, differentiation, or both. We exposed primary rat OPC cultures to 800 nM, 8 µM, or 24 µM of DTG for 24 hours in proliferation medium before switching to differentiation medium without any drug treatment (Figure 4a-b). After 72 hours of differentiation, we observed significantly fewer GalC+ (Figure 4c) and PLP+ (Figure 4d) OLs without a change in DAPI+ cells (Figure 4e) in cultures treated with 24 µM DTG, but not in those treated with either 800 nM or 8 µM DTG. Western blotting (Figure 4f) likewise indicated significantly lower expression of MBP (Figure 4g) and PLP (Figure 4h) myelin proteins compared to DMSO only with 24 µM DTG exposure during proliferation. Therefore, exposure to 24 µM DTG inhibits OL differentiation even when limited to the OPC proliferation stage. To determine the response of OLs to DTG exposure specifically during differentiation, we allowed OPCs to proliferate in the absence of any drug before changing to differentiation medium with 800 nM, 8 µM, or 24 µM doses of DTG (Figure 5a). Immunostaining (Figure 5b) revealed significantly fewer GalC+ (Figure 5c) and PLP+ (Figure 5d) OLs without an effect on DAPI+ cells (Figure 5e) after differentiation exposure to 24 µM DTG, but not 800 nM or 8 µM, similar to that observed with proliferation treatment (Figure 4b-e). In conjunction, Western blotting (Figure 5f) revealed significantly less MBP (Figure 5g) and PLP (Figure 5h) myelin protein expression compared to DMSO only after 24 µM DTG exposure during differentiation. The same effects on OL maturation were observed when DTG exposure was delayed for 24 hours after switching to differentiation medium (Supp. Fig. 3a), showing that 24 µM DTG is sufficient to impair OL development even when limited to later stages of differentiation (Supp. Fig. 3b-e). These data reveal that at high doses, DTG is capable of significantly impairing OL maturation even when exposure is transient during only OPC proliferation or OL differentiation.

### RNA Sequencing Reveals Altered Gene Expression Related to Multiple Cellular Mechanisms in Oligodendroglia After DTG Exposure

**Figure 6.**
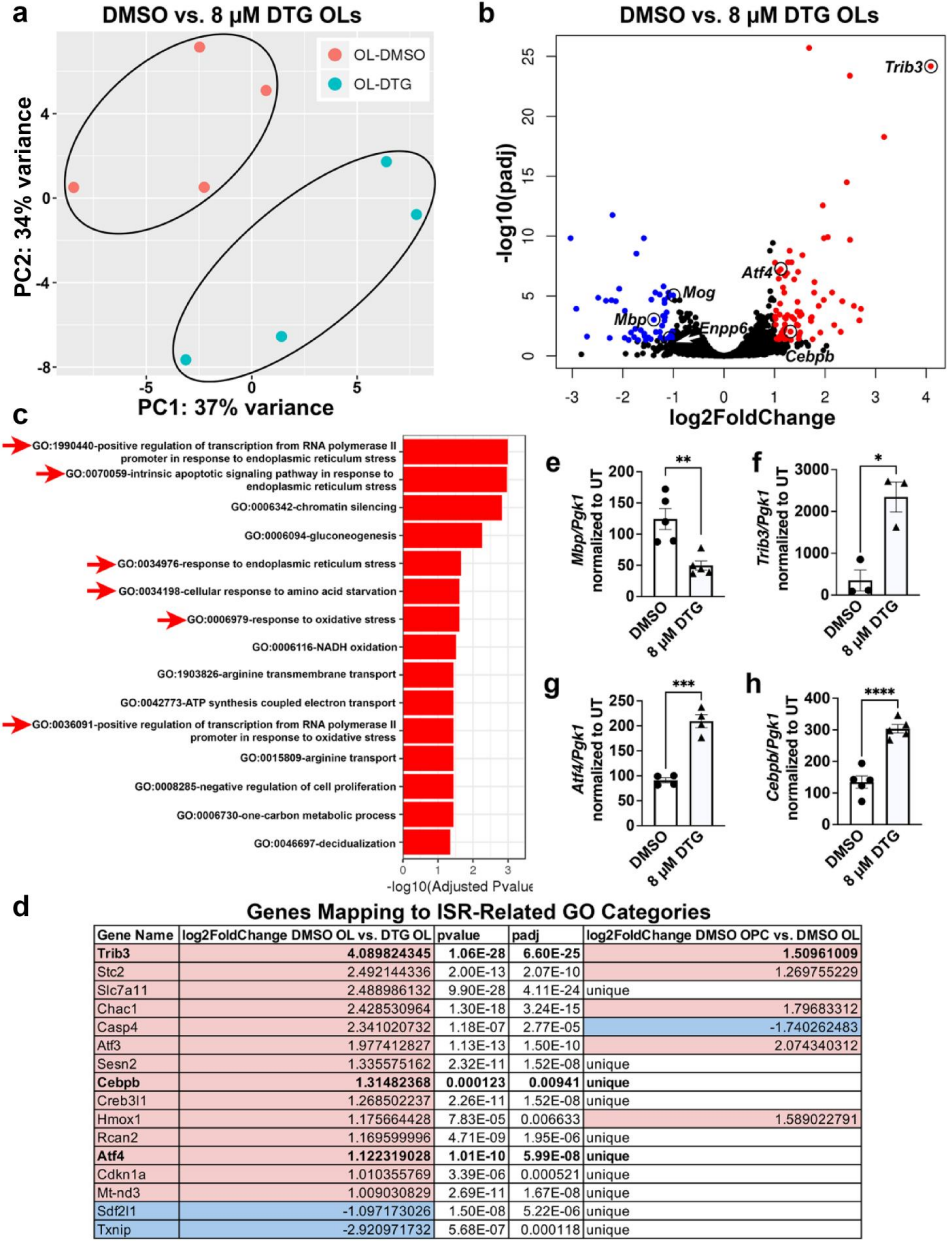
Multistage 8 µM DTG significantly alters gene expression across a variety of cellular mechanisms in differentiating OLs. (a) Principle components (PC) plot of variance between DMSO-treated and 8 µM DTG-treated differentiating OL samples from bulk RNA sequencing, showing clustering of groups, *n* = 4/group. (b) Volcano plot of gene expression in 8 µM DTG-treated OLs vs. DMSO-treated OLs, where red indicates significantly upregulated genes and blue indicates significantly downregulated genes. Particular genes of interest are labeled. (c) Significantly altered gene ontology (GO) categories in 8 µM DTG-treated OLs vs. DMSO-treated OLs. Red arrows highlight GO categories directly related to ISR (ER stress, oxidative stress, amino acid starvation). (d) List of significantly altered genes mapping to ISR-related GO categories with their log2FoldChange in DMSO OLs vs. 8 µM DTG OLs and log2FoldChange in DMSO OPCs vs. DMSO OLs for comparison, *n* = 4/group. Pink = upregulated, blue = downregulated in 8 µM DTG OLs compared to DMSO OLs. qRT-PCR quantification shows significantly decreased expression of *Mbp***(e)** and increased *Trib3* (f), *Atf4* (g), and *Cebpb* (h) in 8 µM DTG-treated vs. DMSO-treated OLs, *n* = 3-5/group. Unpaired *t*-test to compare 8 µM DTG to DMSO. *p < 0.05, **p < 0.01, ***p < 0.001, ****p < 0.0001.

In order to identify potential mechanisms responsible for the observed deficits in OL differentiation, we performed bulk RNA sequencing of primary rat OL cultures treated with 8 µM DTG, the C_max_ dose, across both proliferation and differentiation. Our data indicate that multistage exposure to this dose of DTG equivalent to plasma C_max_ in humans is sufficient to robustly and significantly impair OL maturation *in vitro* (Figure 2,3). Therefore, we collected whole cell RNA from vehicle-treated OPCs exposed to DMSO or DTG-treated OPCs exposed to 8 µM DTG for 24 hours of proliferation, as well as vehicle-treated OLs exposed to DMSO and 8 µM DTG-treated OLs across our multistage paradigm. Surprisingly, there were no significantly altered RNA transcripts when comparing DTG-treated to DMSO-treated OPCs after 24 hours of proliferation (data not shown). Conversely, principal component analysis showed clustering of DMSO-treated and 8 µM DTG-treated OLs after multistage treatment suggestive of differences between these two groups (Figure 6a). Differential expression analyses revealed 127 differentially expressed genes in OLs with multistage 8 µM DTG exposure compared to vehicle-treated control OLs with both downregulated and upregulated genes (Figure 6b, Supp. Fig. 4, Online Resource 1). GO analysis indicated that these genes were spread across a variety of cellular mechanisms (Figure 6c, Online Resource 2). Specifically, the three most significantly altered GO categories were positive regulation of transcription from RNA polymerase II promoter in response to ER stress, intrinsic apoptotic signaling pathway in response to ER stress, and chromatin silencing. We were also interested to see that several of the GO categories were related to metabolic and mitochondrial function, including gluconeogenesis, cellular response to amino acid starvation, NADH oxidation, and ATP synthesis coupled electron transport. However, the 127 significantly altered transcripts would likely include genes that normally up- or downregulate during differentiation and whose opposite regulation would simply suggest a more OPC-like stage of maturation. For example, we verified that multistage exposure to DTG during differentiation resulted in reduced ectonucleotide pyrophosphatase/phosphodiesterase 6 (*Enpp6*)*, Mbp,* and *Mog* transcripts related to OL maturation and myelin production (Figure 6b, Supp. Fig. 5, Online Resources 1,3), and our qRT-PCR validation confirmed that *Mbp* transcription was significantly decreased in DTG-treated OLs by nearly 75% compared to DMSO-treated OLs (Figure 6e). Therefore, we compared our list of 127 genes to over 4200 differentially expressed genes in differentiated vehicle-treated OLs vs. undifferentiated vehicle-treated OPCs (Online Resources 3,5). OPCs and differentiated OLs showed distinct clustering by principal component analysis (Supp. Fig. 5a). The 4200+ differentially expressed genes included *Enpp6, Mbp, Mog, Mag*, *Plp1*, 2’3′-cyclic nucleotide 3′-phosphodiesterase (*Cnp*), and myelin regulatory factor (*Myrf*), all significantly upregulated myelin-related transcripts in differentiated OLs compared to OPCs (Supp. Fig. 5b). Differentially expressed genes in differentiated OLs mapped to 20 significantly altered GO categories including “intracellular signal transduction,” “protein phosphorylation,” and “brain development” (Supp. Fig. 5c, Online Resource 4). In comparing the differentially expressed genes in OPCs vs. differentiated OLs and DMSO-treated OLs vs. DTG-treated OLs, we found 54 differentially expressed genes that were unique to DTG-exposed OLs, normally unchanged during differentiation (Online Resource 5). We also observed 14 transcripts that were significantly upregulated above the normal increase in expression during differentiation, and 1 transcript that was significantly downregulated below the normal decrease in expression during differentiation (Online Resource 5). We then examined which genes made up our GO analysis hits. Specifically, there were 58 hits across all significant GO categories composed of 36 distinct transcripts. Of these, 18 were unique genes affected by DTG exposure and 9 were upregulated genes significantly higher than normal during differentiation. These genes accounted for 75% of all GO analysis hits despite only making up 21.3% of all significantly differentially expressed genes (Online Resources 2,5).

### DTG Induces Significant Transcriptional Changes in Key Integrated Stress Response Effectors

GO analysis revealed that 6 out of 15 significantly altered GO categories directly related to activation of the ISR, including ER stress, oxidative stress, or amino acid starvation (Figure 6c). These ISR-related GO categories included 24 hits from 16 transcripts, 15 of which were either unique or upregulated genes significantly higher than normal during differentiation (Figure 6d). We were particularly interested to see significant transcriptional upregulation of tribbles pseudokinase 3 (*Trib3*), activating transcription factor 4 (*Atf4*), and Ccaat enhancer binding protein b *(Cebpb),* all of which are critically involved in the ISR (Figure 6b). *Trib3* was the single most highly upregulated transcript in DTG- vs. DMSO-treated OLs. While our sequencing data indicate that *Trib3* is normally upregulated during OL differentiation by approximately 285% (log2FoldChange = 1.51), it is robustly upregulated by approximately 1600% (log2FoldChange = 4.09) in DTG-exposed OLs compared to vehicle-treated OLs (Online Resource 5). *Trib3* was sorted into two GO categories, “response to ER stress” and “intrinsic apoptotic signaling pathway in response to ER stress” (Online Resource 2). When expressed during ER stress, TRIB3 modulates the ISR partially by inhibiting the activity of ATF4, the primary effector of ISR activation (Jousse et al., 2007). Therefore, it was interesting to us that *Atf4* was a uniquely upregulated transcript in response to DTG exposure in OLs, approximately 120% (log2FoldChange = 1.12) higher than in vehicle-treated OLs (Online Resource 5) and was a member of 6 different significantly altered GO categories (Online Resource 2), a higher number than any other particular transcript. These were “positive regulation of transcription from RNA polymerase II promoter in response to ER stress,” “intrinsic apoptotic signaling pathway in response to ER stress,” “gluconeogenesis,” “response to ER stress,” “cellular response to amino acid starvation,” and “positive regulation of transcription from RNA polymerase II promoter in response to oxidative stress.” Finally, we identified *Cebpb* as a transcript of interest that was uniquely upregulated by approximately 150% (log2FoldChange = 1.32) in response to DTG (Online Resource 5) and a member of 3 GO categories (Online Resource 2): “positive regulation of transcription from RNA polymerase II promoter in response to ER stress,” “intrinsic apoptotic signaling pathway in response to ER stress,” and “response to ER stress.” As a binding partner of ATF4, C/EBPβ can modulate transcription of the downstream targets of ATF4. Additionally, C/EBPβ can transcriptionally repress ATF4 by binding to the upstream promoter, while ATF4 binds to the *Cebpb* promoter to induce expression. We have previously implicated the ISR as a mechanism of action by which some ART drugs impair OL maturation, and therefore validated the observed upregulations in *Trib3, Atf4,* and *Cebpb* transcription with qRT-PCR. By qRT-PCR, transcriptional expression of *Trib3, Atf4,* and *Cebpb* was significantly and robustly increased in OLs after DTG multistage exposure by approximately 2000% (Figure 6f), 120% (Figure 6g), and 170% (Figure 6h) respectively. Taken together, our sequencing data reveal that DTG exposure in developing OLs *in vitro* significantly impacts cellular mechanisms critical to appropriate OL maturation, in particular related to the ISR.

### Multistage Treatment with DTG Activates the Integrated Stress Response in Differentiating Oligodendrocytes

**Figure 7.**
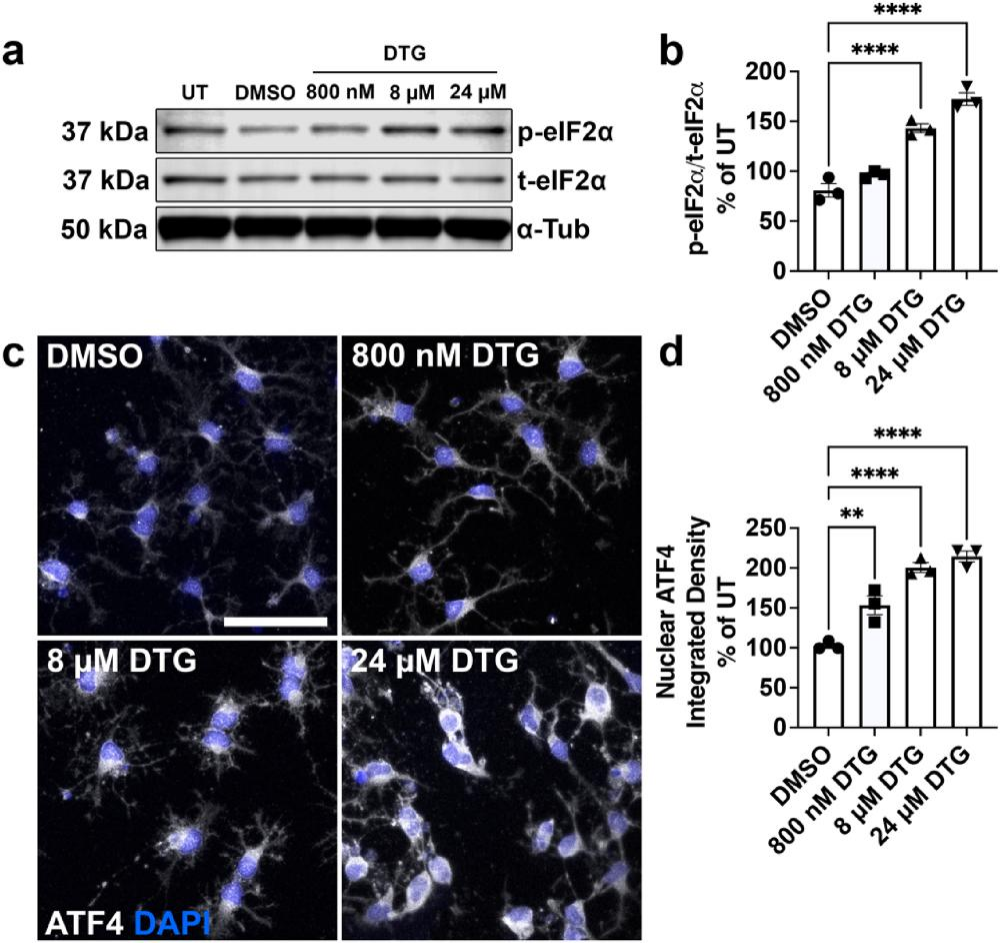
Multistage exposure to DTG activates the ISR in differentiating OLs. (a) Representative immunoblots of phospho-eIF2α and total-eIF2α after multistage exposure to DTG. Densitometry analyses show significantly increased activated eIF2α, (b) after multistage exposure to either 8 µM or 24 µM DTG, *n* = 3/group. (c) Representative images of differentiated OLs after multistage treatment with DTG (800 nM, 8 µM, or 24 µM) and immunostained for ATF4 (white) and DAPI (blue). Scale bar = 50 µm. Quantification of the integrated density of nuclear ATF4, (d) shows significantly increased nuclear ATF4 in differentiated OLs after multistage exposure to all doses of DTG, *n* = 3/group. One-way ANOVA with Dunnett post-hoc test to compare all treatment groups to DMSO. **p < 0.01, ****p < 0.0001.

The ISR is an evolutionarily conserved cytoprotective pathway that is active in eukaryotic cells and significantly contributes to OL homeostasis and proper white matter formation (Chen et al., 2019; Lin et al., 2007). Upon activation, various intracellular and extracellular stressors activate kinases which phosphorylate the eIF2α protein, subsequently resulting in the activation and nuclear translocation of the ATF4 effector protein, which shuts down global protein translation while translating a specific set of mRNAs which aid in bringing the cell back to physiological homeostasis. Previous studies from our research group have implicated the ISR as a mechanism underlying not only ART-drug mediated, but HIV-1 induced CNS damage (Akay et al., 2014; Roth et al., 2021; Roth et al., 2021), and our RNA sequencing data reveal that DTG strongly induces transcriptional changes suggestive of increased ISR activation. To assess ISR activation, we ran Western blots with protein lysates from multistage DTG-treated cells to detect phosphorylated eIF2α protein levels (Figure 7a). Interestingly, we found that exposure to 8 μM and 24 μM DTG concentrations significantly increased the ratios of phosphorylated eIF2α protein to total eIF2α protein after 72 hours of OPC differentiation (Figure 7b). To assess changes in ATF4 activation, we assessed ATF4 nuclear intensity using immunostaining on multistage DTG-treated cells (Figure 7c) and found DTG significantly increased the amount of nuclear-localized ATF4 protein in a concentration dependent manner (Figure 7d). To assess whether ISR activation was specific to multistage DTG exposure, we performed ATF4 immunostaining on OPCs exposed to DTG for 24 hours during proliferation (Supp. Figure 6a) or on differentiated OLs exposed to DTG solely during differentiation (Supp. Figure 6c). In either case, no changes in ATF4 nuclear intensity were observed (Supp. Figure 6b,d), revealing that the mechanism underlying impaired OL maturation with exposure to 24 µM DTG during proliferation or differentiation must be independent of ISR activation, and that ISR activation in OLs is a result of multistage exposure to DTG.

### DTG Acts on Mechanisms Independent of the ISR to Inhibit OL Differentiation

**Figure 8.**
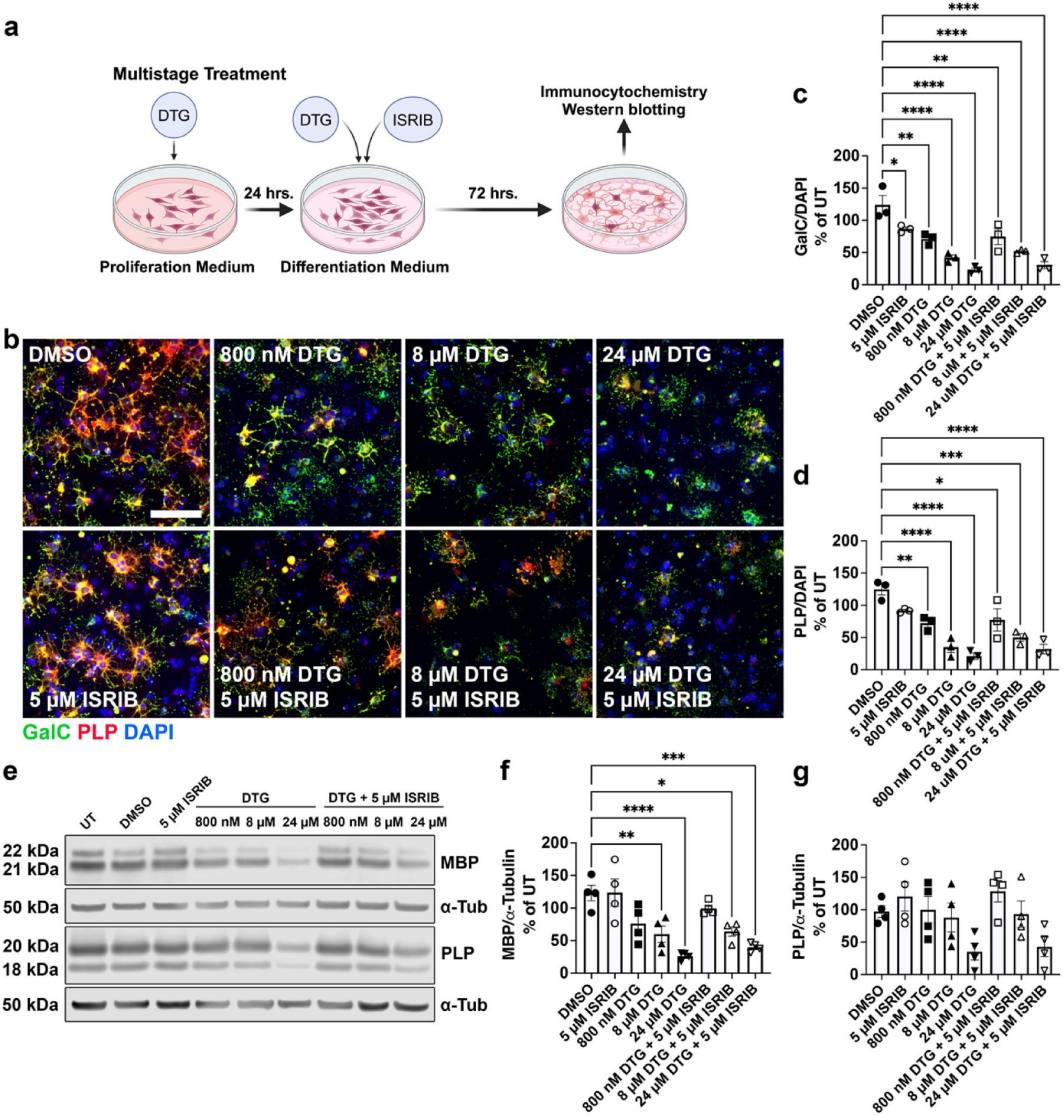
Inhibiting the ISR does not rescue OL differentiation deficits caused by multistage DTG exposure. (a) Diagram of multistage treatment paradigm with DTG and ISRIB during OPC proliferation and OL differentiation. (b) Representative images of differentiated OLs after multistage treatment with DTG (800 nM, 8 µM, or 24 µM) with or without 5 µM ISRIB and immunostained for GalC (green), PLP (red), and DAPI (blue). Scale bar = 75 µm. Quantification of the number of GalC^+^ (c) and PLP^+^ (d) cells shows significantly fewer differentiated OLs after multistage exposure to DTG without rescue by ISRIB, *n* = 3/group. (e) Representative immunoblots of MBP and PLP after multistage exposure to DTG with or without ISRIB. Densitometry analyses do not show rescue by ISRIB in MBP, (f) expression and no effects on PLP, (g) expression, *n* = 4/group. One-way ANOVA with Šídák’s post-hoc test to compare all treatment groups to DMSO and each DTG dose group to DTG + ISRIB. *p < 0.05, **p < 0.01, ***p < 0.001, ****p < 0.0001.

Based on the observed ISR activation in oligodendroglial cultures after multistage DTG treatment, we attempted to rescue the inhibition of OL differentiation induced by multistage 800 nM, 8 µM, or 24 µM DTG exposure by inhibiting ISR activity using the ISR inhibitor (ISRIB) (Sidrauski et al., 2015). Initially, we attempted to add ISRIB concurrently with DTG into our OPC cultures during the 24-hour proliferation treatment; however, this resulted in OPC death (data not shown). Therefore, we treated the OPCs with DTG alone during 24 hours of proliferation before switching to differentiation medium with both DTG and ISRIB for 72 hours (Figure 8a). However, immunostaining for mature OLs (Figure 8b) showed no changes in GalC+ (Figure 8c) or PLP+ (Figure 8d) OL numbers with the addition of ISRIB treatment relative to DTG alone. Likewise, Western blotting for myelin proteins (Figure 8e) did not reveal ISRIB-mediated rescue of reduced MBP (Figure 8f) or PLP (Figure 8g) expression caused by DTG. Despite the strong increase in ISR activation, our data suggest that DTG does not solely act through the ISR to inhibit OL differentiation *in vitro*.

## Discussion

In our study, we demonstrate that DTG exposure across multiple stages of OL development inhibits appropriate differentiation and myelin protein expression. It is striking that a dose as small as 10x lower than human plasma C_max_ significantly impairs OL differentiation when applied across proliferation and differentiation since DTG crosses the placental barrier at about 1.25x maternal plasma levels (Mulligan et al., 2018; Waitt et al., 2019) and is readily prescribed to mothers throughout gestation, during active OPC expansion and early differentiation in the fetus. While our purified OL *in vitro* system does not enable us to measure myelination, this model is excellent for identifying effects on oligodendrocyte differentiation, which occurs robustly during late-stage human fetal gestation. Myelination, by contrast, occurs largely postnatally. However, because OLs continue to differentiate throughout neurodevelopment, our study also provides conclusions that may be applicable beyond gestation. Therefore, our observations may critically aid both the formation of data-driven recommendations for the use of DTG during pregnancy and the improvement of guidelines for the use of DTG-based ART regimens during childhood and adolescence when developmental myelination peaks. These groups may have high vulnerability to white matter changes due to DTG due to continued OPC proliferation and OL differentiation with ongoing myelination through adolescence and into early adulthood.

Due to their highly efficient transfer across the placental barrier, DTG, ABC, and 3TC have the potential to elicit neurodevelopmental side effects in the developing fetus (Best et al., 2006; Chappuy et al., 2004; Moodley et al., 1998; Mulligan et al., 2018; Waitt et al., 2019). ART drug transfer across the BBB in the developing fetus may be more robust than in adults as BBB permeability continues to decrease after birth (Butt et al., 1990; Daneman et al., 2010). Additionally, it is not known whether these drugs accumulate in the white matter during gestational development, as some ART drugs do in the adult population (Ferrara et al., 2020). For these reasons we examined a range of doses for all three drugs (10% C_max_, C_max_, and 300% C_max_) in order to thoroughly elucidate their effects on OL development *in vitro.* It is particularly vital to underscore that even at 300% C_max_, neither ABC nor 3TC had any measurable effect on OL differentiation *in vitro,* while conversely DTG significantly impaired OL maturation at a low dose of 10% C_max_. However, we cannot exclude the possibility that a combination of ABC and 3TC (or DTG in combination with either) would have an additive effect on OL differentiation.

The strongest effects of DTG on OL maturation were observed when any of the three concentrations used were applied during both OPC proliferation and OL differentiation, while 24 µM DTG was sufficient to impede OL maturation if applied only during proliferation or differentiation. Our data therefore reveal that there is an additive effect of exposure to DTG during multiple stages of OL development. These data are particularly important as treatment of pregnant women with DTG exposes both proliferating OPCs and differentiating OLs during gestation. Although exposure to 800 nM or 8 µM DTG during OPC proliferation additively inhibits OL maturation with exposure to DTG during differentiation, no transcriptional changes were observed in OPCs exposed to 8 µM DTG for 24 hours. These findings suggest that DTG may act on cellular mechanisms outside transcription during OPC proliferation and may then induce significant transcriptional changes in differentiating OLs that additively inhibit OL maturation at lower doses. Additionally, we observed that 24 µM DTG impairs OL maturation even when only applied during proliferation, which suggests the changes occurring in proliferating OPCs exposed to 24 µM DTG have a long-term effect on their ability to differentiate without affecting proliferation itself. It is also key to note that 24 µM DTG similarly impairs OL maturation when applied only during differentiation, or even when delayed until late differentiation. Collectively, our data reveal that 24 µM DTG negatively affects OL maturation even when exposure is limited to distinct phases of OL development. These data may be important to consider as some ART drugs accumulate in the white matter of the brain (Ferrara et al., 2020) and even short-term exposure to a high concentration of DTG impairs OL production.

Although multistage treatment with DTG results in strong activation of the ISR in a dose-dependent manner, treating only during proliferation or differentiation with a high dose of DTG reduces OL differentiation without concurrent ISR activation. Interestingly, while other ART drugs such as elvitegravir impair OL differentiation via ISR activation (Roth et al., 2021), our data using ISRIB to inhibit the ISR suggests that DTG activates other mechanisms in conjunction with the ISR to inhibit OL maturation despite its strong activation. It is possible that ISRIB rescues impaired OL differentiation in the presence of elvitegravir but not DTG, even though both drugs elicit ISR activation, because OL maturation is strongly inhibited by elvitegravir exposure solely during differentiation. As such, inhibition of the ISR need only occur during differentiation to rescue elvitegravir-induced OL differentiation deficits, while DTG may act to prime ISR activation during OPC proliferation when ISRIB cannot be utilized without killing the cells. Alternatively, the difference in these two integrase inhibitors may be activation of other mechanisms by DTG contributing to impaired OL differentiation, such as mitochondrial stress. The toxic effects of ISRIB exposure on OPCs may be due to its inhibition of eIF2α, which has numerous targets in the ISR. One method to circumvent this limitation would be to use anti-sense oligonucleotides to inhibit *Atf4* or other significantly altered ISR effectors from our RNAseq data. While outside of the scope of this study, such experiments would demonstrate whether ISR activation, including during OPC proliferation, is integral to DTG impairment of OL differentiation. Regardless, we cannot exclude the possibility that ISR activation additively contributes to the differentiation deficit we observed. Additionally, it is important to note that ISR activation may have other deleterious effects on OL function, including myelin wrapping and maintenance, that we did not investigate in our *in vitro* models.

There are, of course, numerous possible mechanisms of interest that might contribute to the impairment of OL differentiation. In other cell types, DTG has been shown to modulate calcium signaling via increasing uptake of extracellular calcium (Al Mamun Bhuyan et al., 2016), cause mitochondrial dysfunction and an increase in reactive oxygen species (George et al., 2021), and impair lysosomal function and autophagy (Hui et al., 2021). Published studies show that calcium signaling and normal mitochondrial and lysosomal function are all critical for OL development and myelination (Gregorio et al., 2024; Paez & Lyons, 2020; Schoenfeld et al., 2010). Indeed, our lab previously demonstrated that another INSTI ART drug, bictegravir, as well as two PI drugs, saquinavir and darunavir, all result in lysosomal deacidification that blocks the progression of OL development (Festa et al., 2021; 2023; Long et al., 2025). Future studies will be needed to identify the mechanism(s) responsible for DTG-induced OL maturation deficits and their interactions with the ISR.

We observed numerous significant transcriptional changes indicative of ISR activation in OLs exposed to 8 µM DTG during both proliferation and differentiation. We were particularly interested in *Trib3, Cebpb,* and *Atf4* as they have interconnected roles in the ISR. Both TRIB3 and CEBP/β are binding partners for ATF4 that modulate expression of ATF4 transcriptional targets. When ATF4 is activated, it promotes transcription of *Trib3*, which in turn negatively regulates the transcriptional activity of ATF4. CEBP/β interacts with ATF4 to modulate ATF4 transcriptional activity, and both CEBP/β and ATF4 have been shown to bind to the *Trib3* promoter region (Carraro et al., 2010). Interestingly, *Trib3* was the most significantly upregulated gene by fold-change in mature OLs exposed to DTG. Previous work has demonstrated upregulation of TRIB3 occurs in OLs prior to spontaneous CNS demyelination in young adult *Dmy* rats with an autosomal recessive mutation causing mitochondrial dysfunction, OL hypertrophy, and OL death, and this increased TRIB3 expression also associates with increased oxidative stress (Shimotsuma et al., 2016). The remarkable upregulation of *Trib3* by 2000% in response to DTG exposure during OL differentiation may strongly reduce ATF4 transcriptional activity, even when *Atf4* expression is upregulated and ATF4 is translocated to the nucleus as we observed. In this case, it may also explain why our attempted rescue of OL maturation with ISRIB was ineffective – perhaps ISR activation is already adequately suppressed by TRIB3 and another mechanism related to TRIB3 is responsible for impaired OL differentiation. Alternatively, TRIB3 has been shown to inhibit the protein kinase B (AKT)/mechanistic target of rapamycin (mTOR) intracellular signaling pathway in other cell types (Saltykova et al., 2021; Zhang et al., 2019). Published data show that activation of AKT/mTOR signaling is necessary for appropriate OL differentiation and developmental myelination (Tyler et al., 2009; Wahl et al., 2014). Therefore, TRIB3-mediated inhibition of AKT/mTOR signaling would very likely negatively impact OL maturation.

Our work directly addresses a highly relevant clinical concern for PWH, particularly pregnant women. The positive impact of ART, which has effectively extended the lifespan of PWH to that of those living without infection by suppressing viral replication, is counterbalanced by potential effects of long-term ART use on brain development and function. Duration of ART use correlates with severity of white matter abnormalities in the brain of PWH on ART that are strongly associated with cognitive dysfunction (Jernigan et al., 2011; Underwood et al., 2017). However, limited clinical data is available on the impact of exposure to ART during gestation when OPC populations greatly expand, or during critical phases of developmental and adolescent myelination when ART exposure may have unintended side effects on white matter health. Along with the estimated 1.3 million PWH who become pregnant each year, there are approximately 2.4 million children and adolescents living with HIV worldwide. Both of these groups may be impacted by ART regimens that negatively affect white matter development, such as those containing DTG that impair OL maturation as suggested by our data. Insufficient developmental differentiation of OLs is associated with hypomyelination, which in turn impairs neurological function. Given the necessity of using ART drugs during pregnancy to prevent vertical transmission of HIV, it will be critical for future work to elucidate which ART drugs impact oligodendroglial development and ongoing neurodevelopment in prenatally-exposed children and the mechanisms by which they do so. Importantly, it is again critical to note that neither ABC nor 3TC exhibited any measurable effects on OL maturation *in vitro*, and indeed other select ART drugs similarly do not impact OL development (Jensen et al., 2015; Roth et al., 2021) which suggests prioritization of studies to determine if drugs that do not affect white matter development are also effective in blocking HIV replication and transmission. Our study provides data for evidence-based decisions for clinical trial design to study ART regimens used to treat pregnant women and children with HIV, maximizing viral suppression, while minimizing impact on CNS development. Our hope is that this current study and future research will enable identification of the best ART regimens for preventing HIV replication and transmission with the fewest number of neurobiological side effects.

## Supplementary Material

Online Resource 3 Significant DEGs OPCs DMSO vs OLs DMSO Raw Data.xlsx

Online Resource 2 GO Analysis OL DMSO vs OL DTG Significant GO Categories.xlsx

Online Resource 5 Significant DEGs OL DMSO vs OL DTG Comparison to OPC DMSO vs OL DMSO Sorted and Color Coded Data.xlsx

Online Resource 4 GO Analysis DMSO OPC vs DMSO OL Significant GO Categories.xlsx

Online Resource 1 Significant DEGs OL DMSO vs OL DTG Raw Data.xlsx

Manuscript Supplemental Figures Revised.docx

## Data Availability

All raw and metadata associated with bulk RNA sequencing and gene expression analyses are publicly available at the Gene Expression Omnibus (GEO) database, accession code GSE290215.
