# Supplementary material for: Exposure to Frontline Antiretroviral Dolutegravir Disrupts Oligodendrocyte Development Across Differentiation Stages
Source: ASN Neuro. 2026 Mar 29;18(1):2647877. doi: 10.1080/17590914.2026.2647877 (PMC13034632; doi:10.1080/17590914.2026.2647877)
Supplement: Manuscript Supplemental Figures Revised.docx [file TASN_A_2647877_SM6520.docx]

**
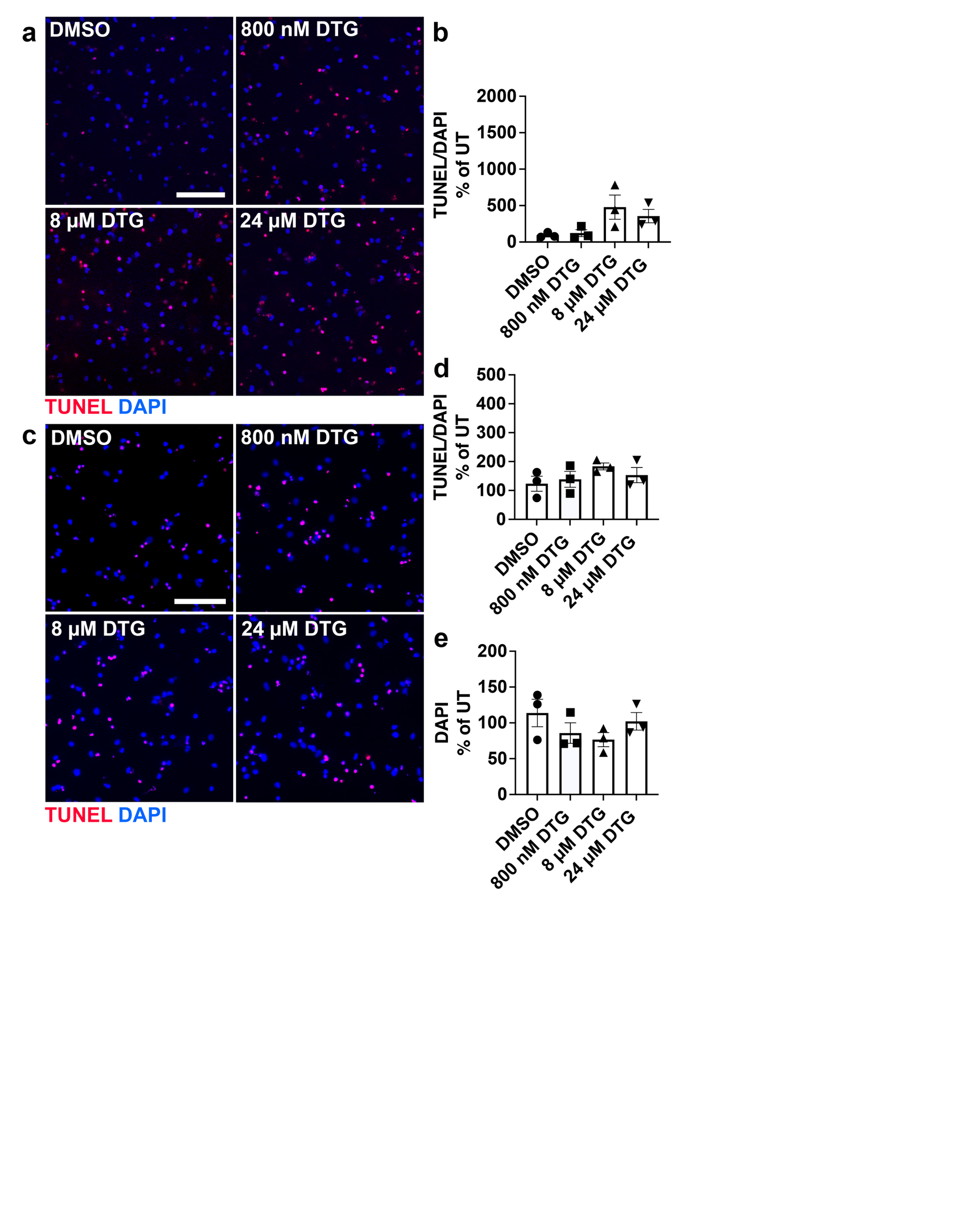
**

**Supplemental Figure 1. Multistage DTG exposure, even when extended, does not alter numbers of apoptotic cells. a)** Representative images of differentiated OLs after multistage treatment with DTG (800 nM, 8 µM, or 24 µM) and stained for TUNEL (red) and DAPI (blue). Scale bar = 75 µm. Quantification of the number of TUNEL^+^ cells **(b)** shows no change in late-stage apoptotic cells after multistage exposure to DTG, n = 3/group. **(c)** Representative images of differentiated OLs after an additional 48 hours of exposure to DTG (800 nM, 8 µM, or 24 µM) and stained for TUNEL (red) and DAPI (blue). Scale bar = 75 µm. Quantification of the number of TUNEL^+^ cells **(d)** shows no change in late-stage apoptotic cells even after extended DTG exposure, n = 3/group. **e)** Quantification of the total number of DAPI^+^ cells is unchanged even after extended exposure to 800 nM, 8 µM, or 24 µM DTG, n = 3/group. One-way ANOVA with Dunnett post-hoc test to compare all treatment groups to DMSO.

**
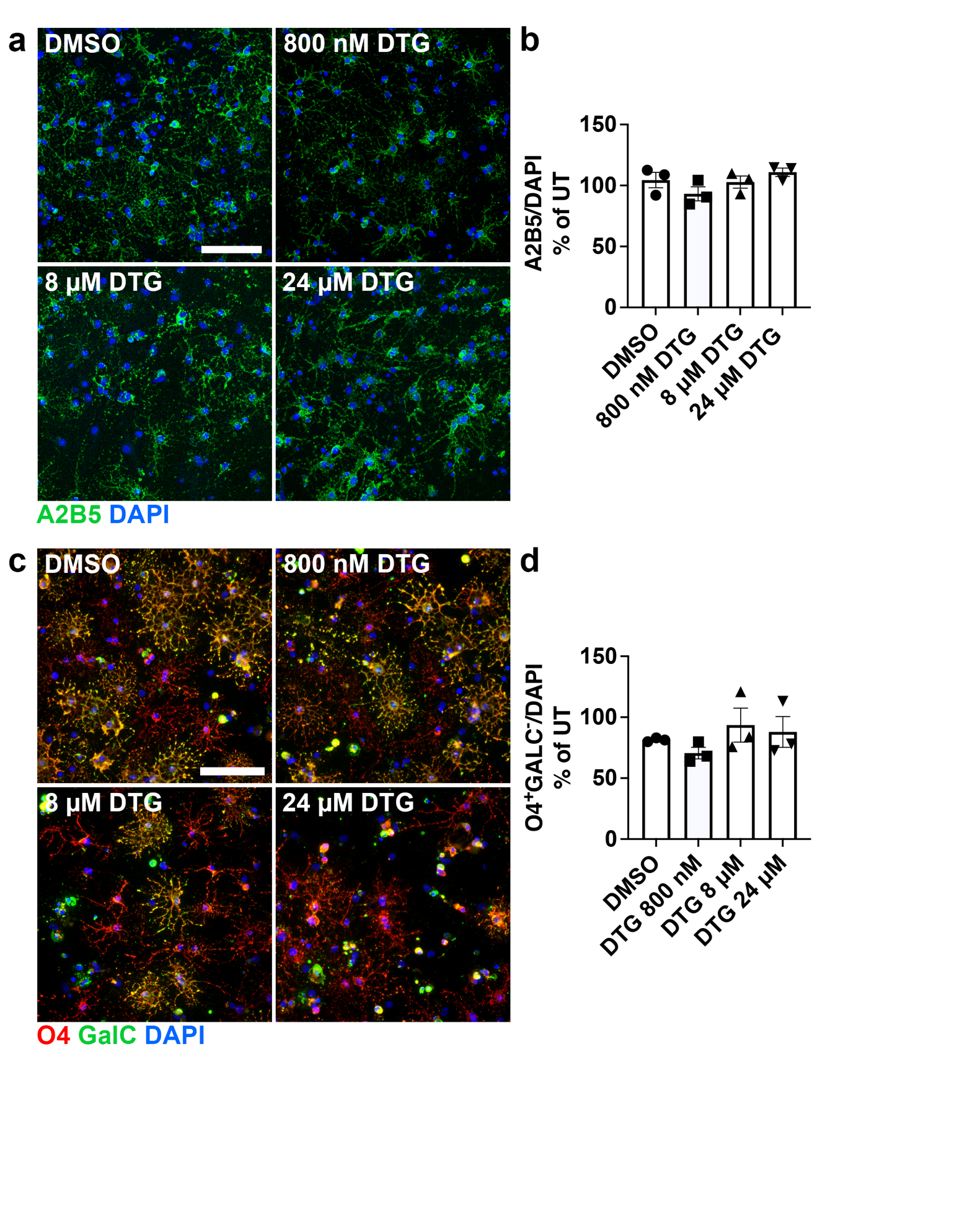
**

**Supplemental Figure 2. Multistage DTG exposure specifically inhibits late-stage OL maturation without affecting OPC or early OL numbers. a)** Representative images of OPCs after multistage treatment with DTG (800 nM, 8 µM, or 24 µM) and stained for A2B5 (green) and DAPI (blue). Scale bar = 75 µm. Quantification of the number of A2B5^+^ cells **(b)** shows no change in OPCs after multistage exposure to DTG, n = 3/group. **c)** Representative images of OLs after multistage treatment with DTG (800 nM, 8 µM, or 24 µM) and stained for O4 (red), GalC (green), and DAPI (blue). Scale bar = 75 µm. Quantification of the number of O4^+^/GalC^-^ early OLs **(d)** shows no change in early OL differentiation after multistage exposure to DTG, n = 3/group. One-way ANOVA with Dunnett post-hoc test to compare all treatment groups to DMSO.

**
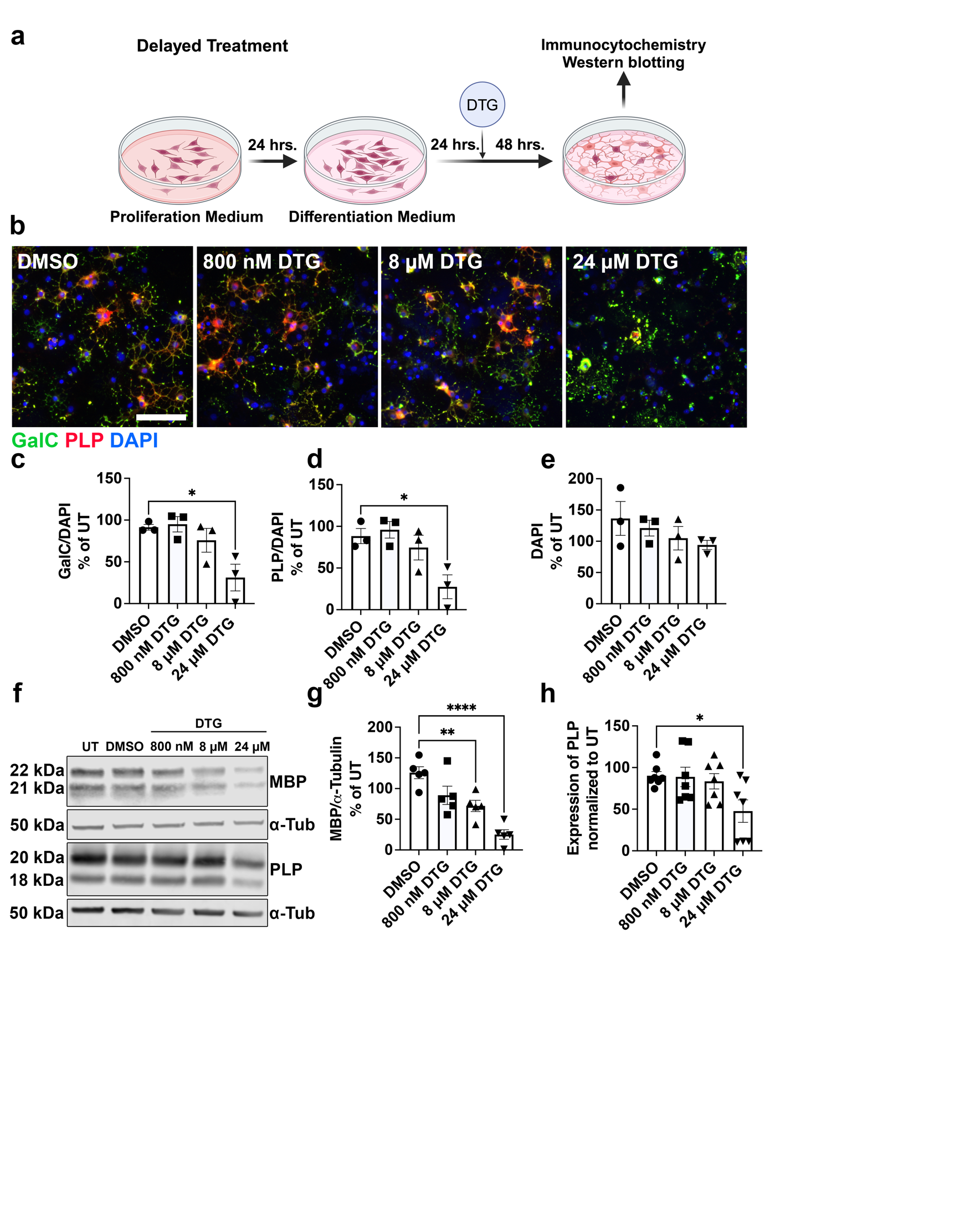
Supplemental Figure 3. Delayed treatment of OLs during differentiation with 24 µM DTG reduces OL maturation. a)** Diagram of delayed differentiation treatment paradigm with DTG. **b)** Representative images of differentiated OLs after delayed differentiation treatment with DTG (800 nM, 8 µM, or 24 µM) and immunostained for GalC (green), PLP (red), and DAPI (blue). Scale bar = 75 µm. Quantification of the number of GalC^+^ **(c)** and PLP^+^ **(d)** cells shows significantly fewer differentiated OLs after delayed differentiation exposure to 24 µM DTG without an effect on total number of DAPI^+^ cells **(e)**, n = 3/group. **f)** Representative immunoblots of MBP and PLP after differentiation exposure to DTG. Densitometry analyses show significantly less MBP **(g)** and PLP **(h)** expression after 24 µM DTG exposure, n = 5-7/group. One-way ANOVA with Dunnett post-hoc test to compare all treatment groups to DMSO. *p<0.05, **p<0.01, ****p<0.0001.

**
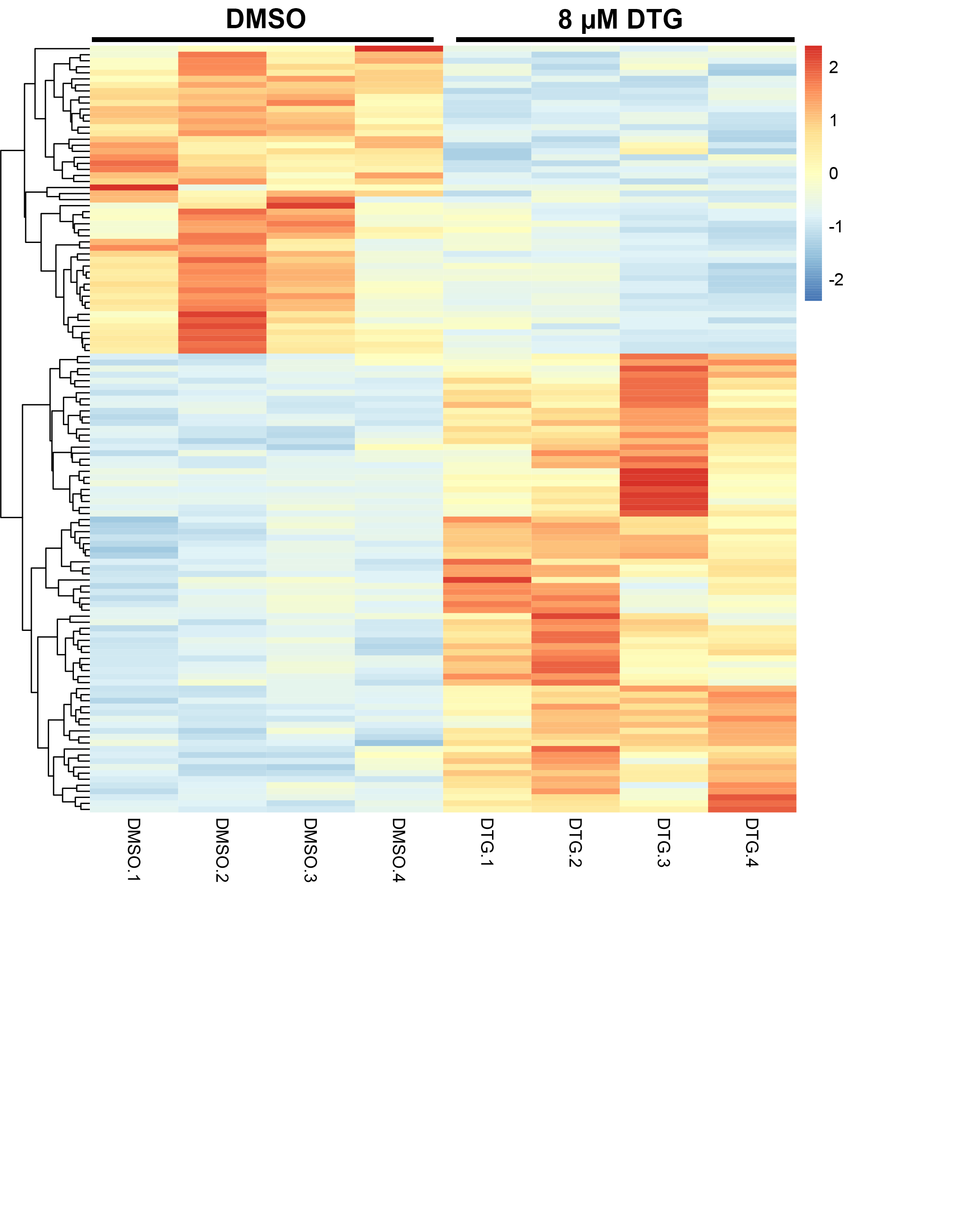
**

**Supplemental Figure 4. DTG shifts gene expression profile in differentiating OLs.** Heat map of all differentially expressed genes in DMSO-treated vs. 8 µM DTG-treated differentiating OLs, showing shifts in gene expression.

**
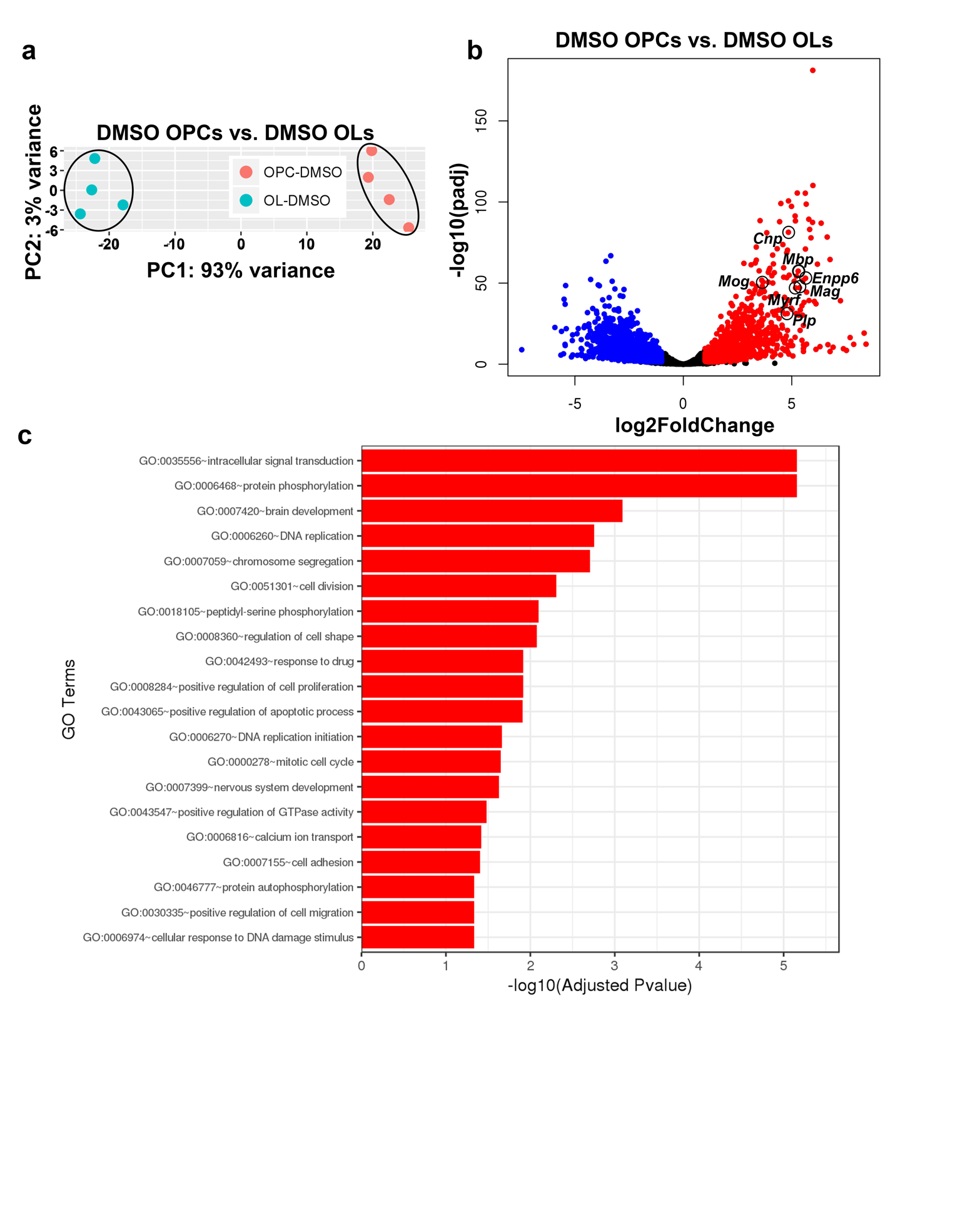
Supplemental Figure 5. OLs undergo significant gene expression changes during differentiation from OPCs. a)** Principle components (PC) plot of variance between DMSO-treated OPCs and DMSO-treated OLs from bulk RNA sequencing, showing clustering of groups, n = 4/group. **b)** Volcano plot of gene expression in OLs vs. OPCs, where red indicates significantly upregulated genes and blue indicates significantly downregulated genes. Myelin-related genes of interest are labeled. **c)** Significantly altered gene ontology (GO) categories in OLs vs. OPCs.

**
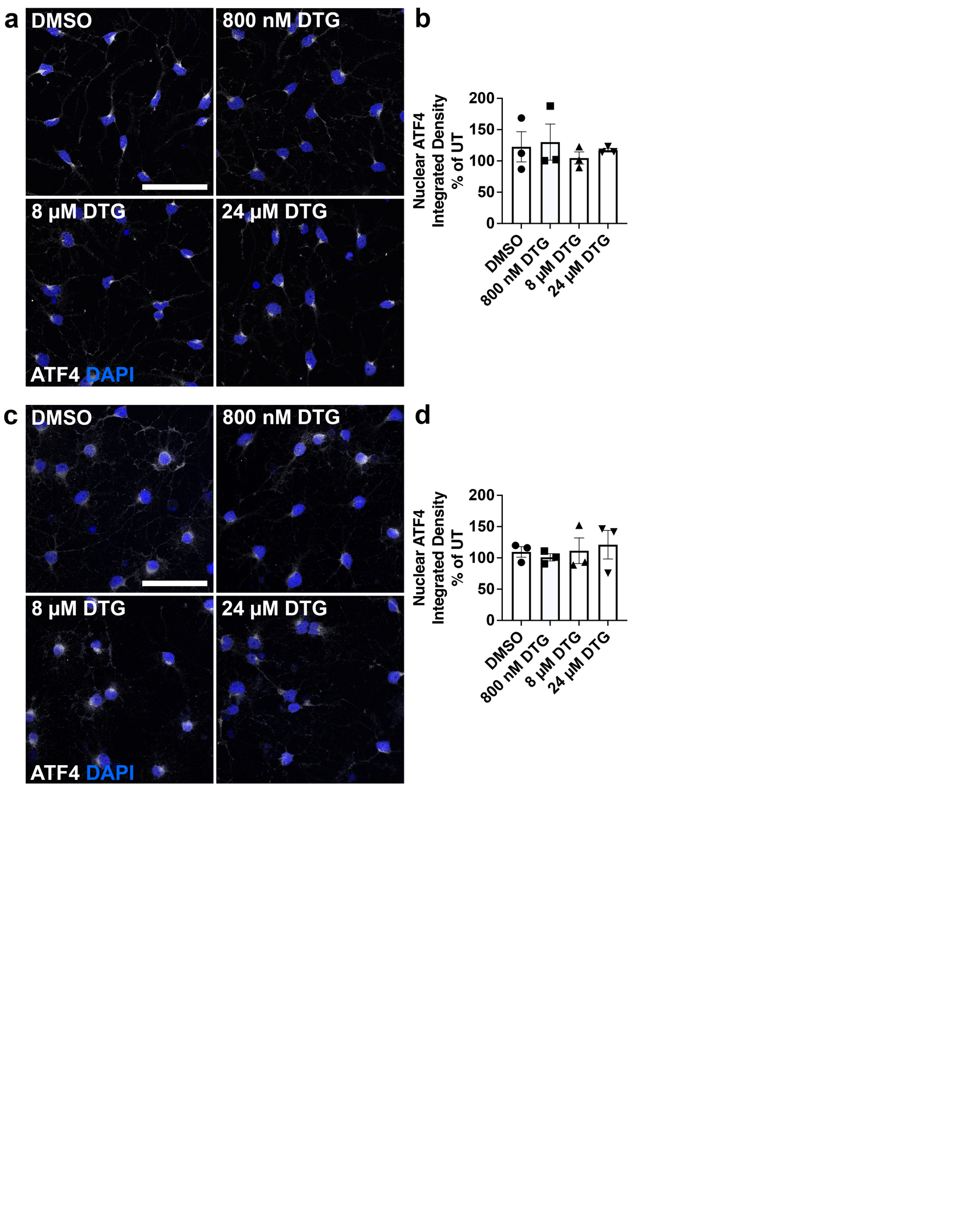
Supplemental Figure 6. Exposure to DTG during either proliferation or differentiation does not activate the ISR in differentiating OLs. a)** Representative images of OPCs after proliferation treatment for 24 hours with DTG (800 nM, 8 µM, or 24 µM) and immunostained for ATF4 (white) and DAPI (blue). Scale bar = 50 µm. Quantification of the integrated density of nuclear ATF4 **(b)** shows unchanged nuclear ATF4 in OPCs after proliferation exposure to all doses of DTG, n = 3/group. **c)** Representative images of differentiated OLs after differentiation treatment with DTG (800 nM, 8 µM, or 24 µM) and immunostained for ATF4 (white) and DAPI (blue). Scale bar = 50 µm. Quantification of the integrated density of nuclear ATF4 **(d)** shows unchanged nuclear ATF4 in differentiated OLs after differentiation exposure to all doses of DTG, n = 3/group. One-way ANOVA with Dunnett post-hoc test to compare all treatment groups to DMSO.
